# A machine learning approach to automatic detection of irregularity in skin lesion border using dermoscopic images

**DOI:** 10.7717/peerj-cs.268

**Published:** 2020-06-29

**Authors:** Abder-Rahman Ali, Jingpeng Li, Guang Yang, Sally Jane O’Shea

**Affiliations:** 1 Faculty of Natural Sciences, Computing Science and Mathematics, University of Stirling, Stirling, UK; 2 National Heart and Lung Institute, Imperial College London, London, UK; 3 Mater Private Hospital, Cork, Ireland

**Keywords:** Machine learning, Dermoscopy, Skin lesion, Melanoma, Segmentation

## Abstract

Skin lesion border irregularity is considered an important clinical feature for the early diagnosis of melanoma, representing the B feature in the ABCD rule. In this article we propose an automated approach for skin lesion border irregularity detection. The approach involves extracting the skin lesion from the image, detecting the skin lesion border, measuring the border irregularity, training a Convolutional Neural Network and Gaussian naive Bayes ensemble, to the automatic detection of border irregularity, which results in an objective decision on whether the skin lesion border is considered regular or irregular. The approach achieves outstanding results, obtaining an accuracy, sensitivity, specificity, and *F*-score of 93.6%, 100%, 92.5% and 96.1%, respectively.

## Introduction

Melanoma is a skin cancer that develops within pigment-producing skin cells called melanocytes. It can be detected clinically due to visual changes, such as a change in shape, color and/or size. Thicker, ulcerated lesions may present due to symptoms such as bleeding. Prognosis is influenced by the early detection and treatment of melanoma. This is reflected in better survival rates for earlier stage disease (Gershenwald et al., 2017). The ABCD rule (Friedman, Rigel & Kopf, 1985) emerged in 1985 by a group of researchers at the New York University as a simple method that physicians, novice dermatologists and non-physicians can use to learn about the features of melanoma in its early curable stage to enhance the detection of melanoma. It is more geared towards the public than the 7-point checklist which was designed for non-dermatological medical personnel. The approach has then been verified by 1992 National Institutes of Health Consensus Conference Report on Early Melanoma, in addition to other studies published at the time (Cascinelli et al., 1987; White, Rigel & Friedman, 1991; Barnhill et al., 1992; McGovern & Litaker, 1992), and is being advertised by the American Cancer Society as a method to help the early medical evaluation of any suspicious pigmented lesions.

The ABCD acronym refers to four parameters: Asymmetry, Border irregularity, Color variegation, and Diameter greater than 6 mm. Such parameters provide simple means for appraisal of pigmented cutaneous lesions that should be assessed by a skin specialist, which would include a dermoscopic evaluation and, excision, where appropriate. The algorithm is designed as a general rule of thumb for the layperson and the primary care physician, as a simple method to detect the clinical features of melanoma. This is intended to help in the detection of thinner melanomas and tends to describe features of the most common melanoma subtype, called superficial spreading melanoma. The rule is not designed to provide a comprehensive list of all melanoma characteristics. The ABCD algorithm has the greatest accuracy when used in combination (i.e., AB, AC, ABC), although melanomas don’t need to to acquire all four features. Referring back to the results of the studies that attempt to document the diagnostic accuracy of the ABCD rule in clinical practice, combining the reliable sensitivity, specificity and adequate inter-observer concordance in the application of the ABCD rule supports the ongoing usage of this rule in clinical practice (Abbasi et al., 2004). The ABCD rule is used in public education on a wide basis and is easy to memorize, and its four features are part of the 7-point checklist. A comparison between the ABCD rule and the 7-point checklist concludes that the ABCD approach has a better sensitivity and a similar specificity (Bishop & Gore, 2008). Evidence afterward has shown that the addition of an *E* criterion for *Evolving* would enhance the ability of early recognition of melanoma (Abbasi et al., 2004).

*Border irregularity* has been reported to be the most significant factor in melanoma diagnosis (Keefe, Dick & Wakeel, 1990). Unlike benign pigmented lesions which tend to present with regular borders, melanoma has an irregular border due to the uneven growth rate (Dellavalle, 2012), the spread of melanocytes in various directions, and the regression of invasion and/or genetic instability of the lesion (Lee & Claridge, 2005).

In this article, we propose a segmentation method to extract the skin lesion, detect its border using the Canny edge detector, derive a vector of irregularity measures to represent the irregularity of the extracted skin lesion border, and eventually use a CNN and Gaussian naive Bayes ensemble to automatically determine whether a lesion is considered regular or irregular based on those measures. The main contributions in our work can be summarized as follows: (i) proposing an image segmentation approach that takes the ambiguous pixels into account by revealing and affecting them to the appropriate cluster in a fuzzy clustering setting; (ii) proposing an objective quantitative measure for representing the skin lesion border irregularity; (iii) using a CNN—Gaussian naive Bayes ensemble for predicting skin lesion border irregularity.

### Related work

Various studies attempting to detect the irregularity of borders in skin lesions and melanoma have been proposed in literature. In Golston et al. (1992), a dermatologist was asked to score 60 skin tumor images as being regular or irregular (regular: 14, irregular: 46). A border was then found using a radial search algorithm (Golston, Moss & Stoecker, 1990), where different windows (i.e., sliding window) are automatically detected in the skin lesion, each of which represents the origin of a radii. Radii are searched for sufficiently high jumps in luminance that also possess sufficiently sustained luminance as those will form the candidate border points (Leondes, 1997). Irregularity is eventualy found using the following formula:

(1) $$I = \displaystyle{{{P^2}} \over {4\rm \pi A}}$$

where *P* and *A* denote the *perimeter* and *area* of the closed boundary, respectively. The perimeter is measured by counting the points on the detected border, and the area is measured by counting the points on and within the border. The authors reached a conclusion that borders with an irregularity index greater than 1.8 were classified as being irregular. Using the proposed algorithm, 42 of the 46 irregular tumors were classified correctly. Of the 14 regular tumors, 8 were classified correctly. Thus, 83.3% of the tumors were classified the same as the dermatologist.

Another study was done by Ng & Lee (1996), where the use of fractal dimensions (FDs) in measuring the irregularity of skin lesion borders is investigated. For each color image, four fractal dimension measures are found: direct FD, vertical smoothing FD, horizontal smoothing FD, and multi-fractal dimension of order two. Those FDs are also calculated on the blue band of the images. After being segmented by a multi-stage method (Lee et al., 1995), 468 melanocytic lesions (not hairy) are used to test the proposed approach. Results show that the multi-fractal method performs the best. Another work where FDa were used is found in Claridge, Smith & Hall (1998).

An automatic approach for analyzing the structural irregularity of cutaneous melanocytic lesion borders was proposed in Lee et al. (1999). The algorithm consists of two stages. In the first *pre-processing* stage, the lesion border is extracted from the skin images after removing the dark thick hair by DullRazor (Lee et al., 1997). In the second stage, the structural shape of the lesion border is analyzed using a proposed measure, namely *sigma-ratio*, which is derived from the scale-space filtering technique with an extended scale-space image. Results show that unlike shape descriptors such as *compactness index* and *fractal dimension* that are more sensitive to texture irregularities than structure irregularities (i.e., don’t provide accurate estimation for the structure irregularity) (Lee & Atkins, 2000), sigma-ratio is considered sensitive to the structural indentations and protrusions. The authors further improved their past work to propose a new border irregularity measure in Lee & Atkins (2000), Lee, McLean & Stella (2003) and Lee & Claridge (2005). The new method works first by locating all indentations and protrusions along the lesion border, and a new *irregularity index* is measured for each indentation and protrusion. Summing up all the individual indices provides an estimation on the overall border irregularity. A new feature was also introduced in the proposed method in that it is able to localize the significant indentations and protrusions.

Aribisala & Claridge (2005) proposed a new measure of border irregularity based on *conditional entropy*, where it was observed that the entropy increases with the degree of irregularity. A total of 98 skin lesions are used in the experiments, of which 16 are melanoma. The results of the proposed measure are compared with the Indentation Irregularity Index (Lee & Claridge, 2005) and show to have a better discriminatory power such that the area under the ROC curve was 0.76 compared to 0.73 for the Indentation Irregularity Index. In particular, the proposed measure gives 70% sensitivity and 84% specificity.

Ma et al. (2010) used *wavelet decomposition* to extract the skin lesion border structure, based on which they would determine whether the lesion is a naevus or melanoma. Using the discrete wavelet transform (DWT), the 1D border is filtered into sub-bands down to level 9 where levels 6–9 (significant levels) have shown to contain information considered best for classifying between melanoma and benign samples. Some statistical and geometrical feature descriptors of border irregularity are extracted at each individual sub-band. A back-projection neural network is used as a classifier which receives a combination of features as input. 25 measurements are formed by applying 6 features in four significant sub-bands, and one feature in a single sub-band. Using a small training set of 9 melanomas and 9 naevi, the best classifier is obtained when the best 13 features are used.

A system was proposed by Jaworek-Korjakowska & Tadeusiewicz (2015), which consists of the following steps: image enhancement, lesion segmentation, border irregularity detection, and classification. To find the border irregularity, the authors translated the border into a function with peaks that indicate the border irregularity. This is achieved by implementing a four step algorithm: (i) computing the bounding box of the segmented skin lesion; (ii) finding the boundary pixels lying on the lines that connect the center of the mass with the vertices; (iii) calculating the distance between the border and the edge of the image, which results in a function with an exact reflection of border irregularities. The signal is smoothed using a Gaussian filter in order to determine the ragged edges; (iv) finally calculating the derivative to find the local maximum points of the function, such that the local maximum is detected when the function crosses the zero point and the slope changes from + to −. The authors used a simple method to measure border irregularity, in which a simple semi-quantitative evaluation method is used to divide the lesion into eight similar parts such that the sharp abrupt cut-off in each part has a score of 1. Thus, a maximum score of 8 is obtained if the whole border is irregular, and a score 0 is obtained if the naevus is round with no ragged borders. As a rule of thumb, melanomas tend to have scores 4–8 (Argenziano et al., 2000). This was tested on 120 skin lesion cases with border irregularity less than 3 and 180 skin lesion cases with border irregularity greater than 4, and the proposed approach achieved a 79% accuracy.

As opposed to the studies mentioned above, we emphasize the use of machine learning in detecting (automatically) skin lesion border irregularity, in addition to proposing a comprehensive irregularity measure that combines different irregularity aspects. In particular, we develop a novel automated approach for detecting skin lesion border irregularity, in which we propose a segmentation method to extract the skin lesion from the image, followed by Canny edge detection to detect the borders of the skin lesion, and then eventually using this border to obtain a vector of measures that represent the irregularity of the skin lesion border. A CNN and Gaussian naive Bayes ensemble is then used to provide a decision on the irregularity of the given skin lesion border.

**Figure 1:**
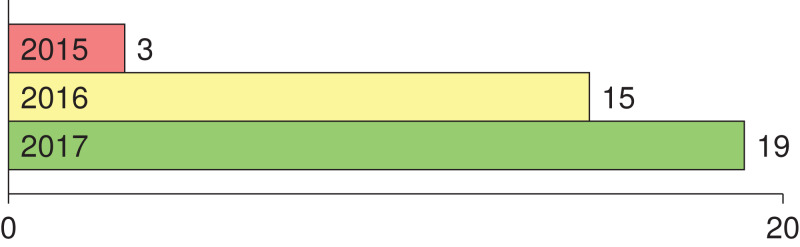
Number of main published papers using deep learning in melanoma detection in the period 2015–2017.

Numerous studies utilizing deep learning (i.e., CNN) for melanoma detection have been recently proposed in literature. To the best of our knowledge, the earlier attempts in applying deep learning to melanoma detection were proposed in 2015 in Codella et al. (2015), Yoshida & Iyatomi (2015) (in Japanese) and Attia et al. (2015). Tracking the number of main studies where deep learning was employed for melanoma detection in the period 2015–2017 (Fig. 1), we find that the number has been increased. Since 2018 the number has been harder to track due to the dramatic number of papers published on the topic. This list shows the main papers published in 2016: Kawahara, BenTaieb & Hamarneh (2016), Kawahara & Hamarneh (2016), Premaladh & Ravichandran (2016), Jafari et al. (2016b), Nasr-Esfahani et al. (2016), Sabbaghi, Aldeen & Garnavi (2016), Pomponiu, Nejati & Cheung (2016), Majtner, Yildirim-Yayilgan & Yngve Hardeberg (2016), Cıcero, Oliveira & Botelho (2016), Menegola et al. (2016), Demyanov et al. (2016), Jafari et al. (2016a), Sabouri & GholamHosseini (2016), Salunkhe & Mehta (2016), Karabulut & Ibrikci (2016); the main papers published in 2017 are as follows: Yu et al. (2017a), Codella et al. (2017b), Esteva et al. (2017), Menegola et al. (2017), Lopez et al. (2017), Kwasigroch, Mikołajczyk & Grochowski (2017a), Mirunalini et al. (2017), Elmahdy, Abdeldayem & Yassine (2017), Attia et al. (2017), Yuan, Chao & Lo (2017), Zhang (2017), Raupov et al. (2017), Bozorgtabar et al. (2017), Liao & Luo (2017), Burdick et al. (2017), Yu et al. (2017b), Kwasigroch, Mikolajczyk & Grochowski (2017b), Georgakopoulos et al. (2017), Monika & Soyer (2017).

Table 1 highlights 13 papers we choose from the ones published in the period 2015–2017 along with their years of publication, implementation frameworks used, and the details of the datasets utilized (i.e., size, image type). Papers chosen were those that had sensitivity and specificity values demonstrated, and the main deep learning approach used in melanoma detection explained. Five studies use a dataset size less than 1,000 images (Premaladh & Ravichandran, 2016; Jafari et al., 2016b; Nasr-Esfahani et al., 2016; Sabbaghi, Aldeen & Garnavi, 2016; Pomponiu, Nejati & Cheung, 2016), two studies use more than 1,000 images (Kawahara, BenTaieb & Hamarneh, 2016; Kawahara & Hamarneh, 2016), five studies use more than 2,000 images (Codella et al., 2015, 2017b; Majtner, Yildirim-Yayilgan & Yngve Hardeberg, 2016; Yu et al., 2017a; Attia et al., 2017), and one study uses more than 100,000 images (Esteva et al., 2017).

**Table 1:** Selected papers using deep learning for melanoma detection in the period 2015–2017.

Paper	Year	Framework	Dataset	Size	Imagetype
Codella et al. (2015)	2015	Caffe	ISIC	2,024	Dermoscopy
Kawahara, BenTaieb & Hamarneh (2016)	2016	Caffe	Dermofit Image Library	1,300	Digital
Kawahara & Hamarneh (2016)	2016	Caffe	Dermofit Image Library	1,300	Digital
Premaladh & Ravichandran (2016)	2016	NA	images collected from various repositories	992	Dermoscopy, digital
Jafari et al. (2016b)	2016	MATLAB and Caffe	Dermquest	126	Digital
Nasr-Esfahani et al. (2016)	2016	NA	MED-NODE	170	Digital
Sabbaghi, Aldeen & Garnavi (2016)	2016	NA	The National Institutes of Health, USA	814	Dermoscopy
Pomponiu, Nejati & Cheung (2016)	2016	Caffe	DermIS, Dermquest	399 (enlarged to 10K)	Digital
Majtner, Yildirim-Yayilgan & Yngve Hardeberg (2016)	2016	MatConvNet	ISIC	2,624	Dermoscopy
Yu et al. (2017a)	2016	Caffe	ISIC	2,624	Dermoscopy
Codella et al. (2017b)	2017	Theano, Lasange, Nolearn, Caffe	ISIC	2,624	Dermoscopy
Codella et al. (2017b)	2017	Tensorflow	ISIC, Dermofit Image Library, Stanford Medical Center	129,450	Dermoscopy, digital
Attia et al. (2017)	2017	NA	ISIC	2,624	Dermoscopy

**Figure 2:**
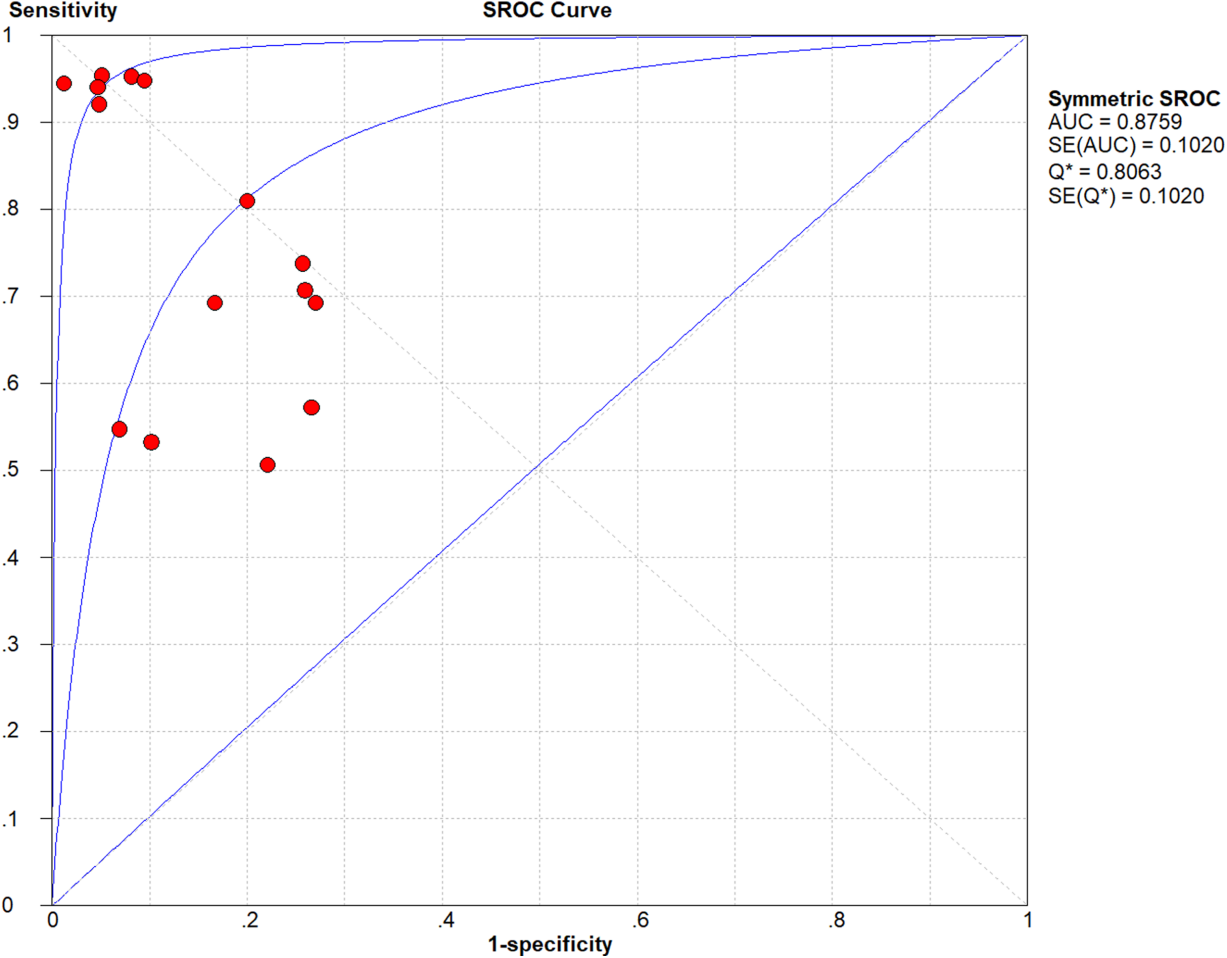
Summary receiver operating characteristic (SROC) of the chosen studies (plotted using Meta-DiSc 1.4: http://www.hrc.es/investigacion/metadisc_en.htm).

The lowest and highest sensitivity values reported in the chosen studies are 0.51 and 0.95, respectively, with a pooled sensitivity of 0.76. The lowest and highest specificity values reported on the other hand are 0.73 and 0.99, respectively, with a pooled specificity of 0.86. The pooled DOR (diagnostic odds ratio) evaluates to 12.95; a small increase in the likelihood of the disease (i.e., + = 3.82) and a small decrease in the likelihood of the disease (i.e., − = 0.41) have also been noticed. The higher the DOR the better the test. A test provides no diagnostic evidence if DOR =1, while a test with DOR > 25 provides strong diagnostic evidence and a test with DOR > 100 provides a convincing diagnostic evidence. Using deep learning for melanoma detection thus shows poor diagnostic performance as evidenced by the DOR (12.95), which depicts that the odds of a positive test result is 12.95 times greater for someone with melanoma than without melanoma. This finding is confirmed by the likelihood ratio, where + = 3.82 means that the positive malignancy (i.e., melanoma) is 3.82 times more common in patients with melanoma than in those without melanoma. In other words, the patient’s positive test result would be 3.82 times more likely to be seen in someone with melanoma than in someone without melanoma. On the other hand, − = 0.41 shows that a negative malignancy (i.e., benign) is 0.41 times more likely to be seen in patients with melanoma than in without melanoma. This poor diagnostic performance could be due to different factors like the small datasets and the quality of images used. The general accuracy of the tests is considered *good* referring to their AUC = 0.8759 value (area under the receiver operating characteristic—ROC). The accuracy of the tests improve when the summary receiver operating characteristic (SROC) curve (Fig. 2) moves to the top-left corner. That is, towards the point (1, 0) of the graph. Results also show good accuracy in terms of pooled sensitivity (0.76) and pooled specificity (0.86). The reason for the extra test results (red circles) shown in the figure (i.e., >13) is due to the fact that some studies (Codella et al., 2015, 2017b) include more than one experimental result in their work.

While most studies presented in literature that use deep learning in melanoma detection focus on training the neural network on the original image as a way to extract different features and eventually come up with a classification (i.e., melanoma vs. benign), we use deep learning (i.e., CNN) to learn and detect/classify *fine* structures of the skin lesion image, which is border irregularity in this work. As opposed to papers presented in literature, we train the CNN on the skin lesion segmentation result and on the extracted lesion border, and not directly on the original image which would otherwise be very complex to detect such fine structure.

## Methodology

**Figure 3:**
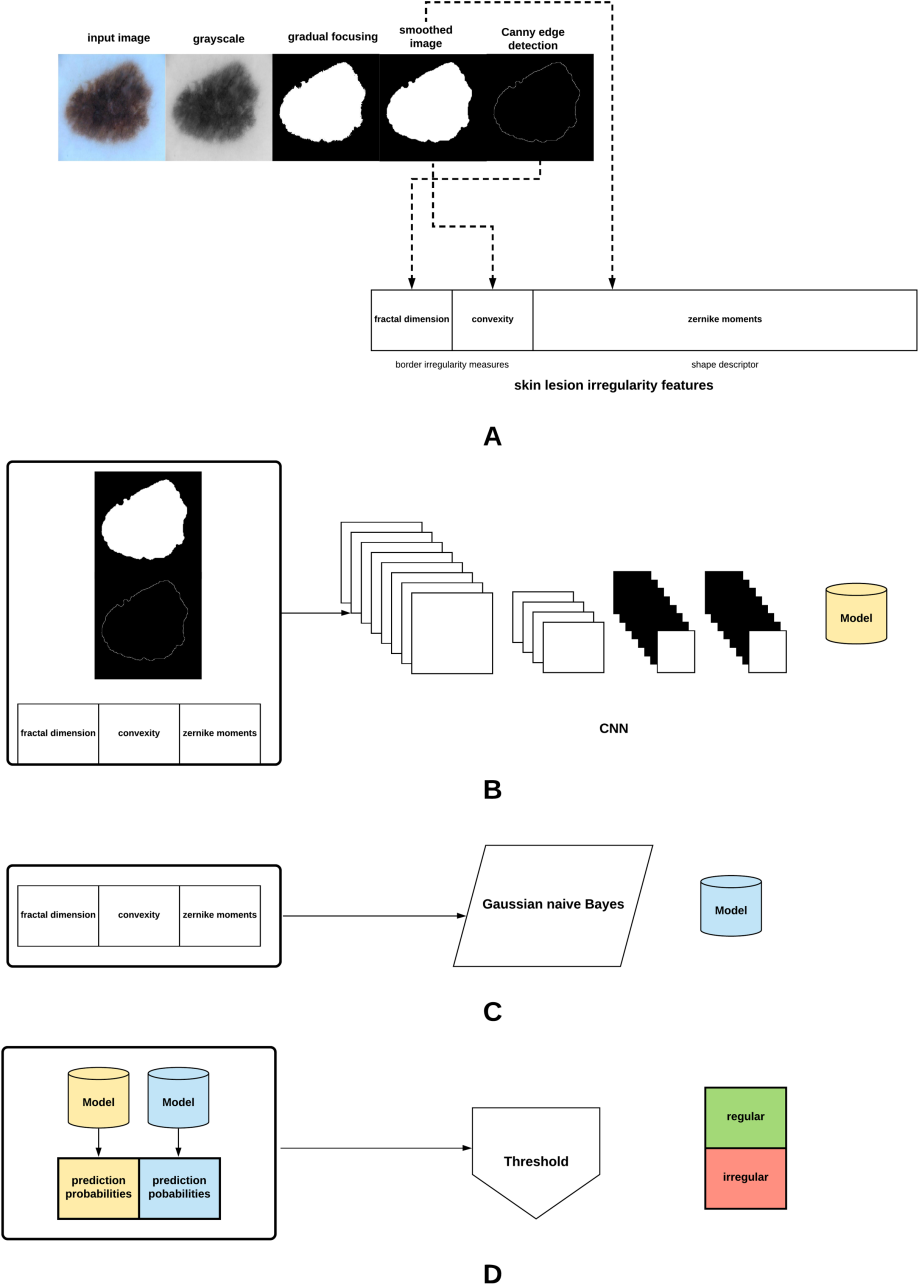
Proposed approach: (A) The skin lesion image is firstly converted to grayscale, after which the skin lesion is segmented and smoothed, lesion border (edge) detected, and the lesion irregularity measure derived. (B) A CNN is trained on the smoothed segmented image, skin lesion border, and the skin lesion irregularity measure. (C) A Gaussian naive Bayes is trained on the irregularity measure. (D) The generated models are used to predict class probabilities, and a threshold is eventually used to determine the final decision (regular or irregular skin lesion border).

The proposed method in this article is summarized in Fig. 3. The different parts of the method will be explained in the subsequent sections.

### Skin lesion extraction

**Figure 4:**
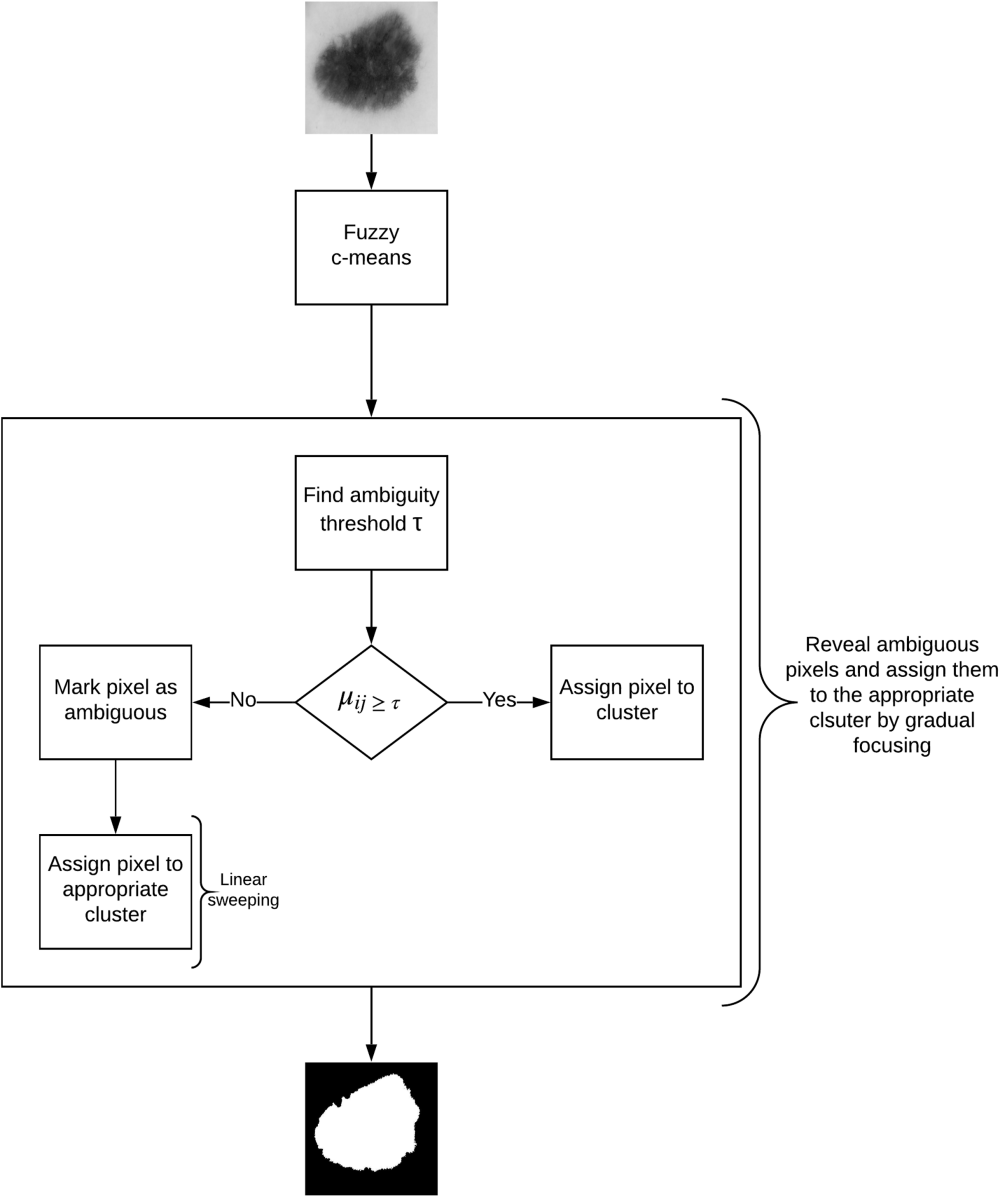
Defuzzification by gradual focusing.

In this article, we propose an image segmentation method for skin lesion extraction which is depicted in Fig. 4. The approach consists of the following main components: (1) fuzzy c-means clustering (2) measuring the optimum threshold (inter-cluster threshold) that distinguishes between the ambiguous and non-ambiguous pixels (3) revealing the ambiguous pixels (4) local treatment of the ambiguous pixels, and (5) final segmentation. This method combines our approaches proposed in our earlier work for liver lesion extraction (Ali et al., 2015, 2016).

Let $X = \left\{ {{x_1},\ldots ,{x_i},\ldots,{x_n}} \right\}$ be the set of $n$ objects (i.e., pixels), $f({x_1},{y_1}),\ldots ,f({x_i},{y_j}):i \in \left[ {1,\ldots ,m} \right];j \in \left[ {1,\ldots ,n} \right]$, and $V = \left\{ {{v_1},\ldots ,{v_i},\ldots ,{v_c}} \right\}$ be the set of *c* centroids in a *p*-dimensional feature space. In fuzzy c-means (FCM), *X* is partitioned into *c* clusters by minimizing the objective function *J*:

(2) $$J = \sum\limits_{j = 1}^n \sum\limits_{i = 1}^c {\left( {{u_{ij}}} \right)^m}{\left\| {{x_j} - {v_i}} \right\|^2}$$

where $1 \le m \le \infty$ is the *fuzzifier* (also called the *fuzzy weighting exponent*), ${v_i}$ is the *i*th centroid corresponding to cluster ${C_i}$, ${u_{ij}} \in \left[ {0,1} \right]$ is the fuzzy membership of ${x_j}$ to cluster ${C_i}$, and $\left\| . \right\|$ is the distance norm, such that:

(3) $${v_i} = \displaystyle{1 \over {{n_i}}}\sum\limits_{j = 1}^n {\left( {{u_{ij}}} \right)^m}{x_j}\quad {\rm where}\quad {n_i} = \sum\limits_{j = 1}^n {\left( {{u_{ij}}} \right)^m}$$

and

(4) $${u_{ij}} = \displaystyle{1 \over {\sum\nolimits_{k = 1}^c {{{\left( {\displaystyle{{{d_{ij}}} \over {{d_{kj}}}}} \right)}^{\textstyle{2 \over {m - 1}}}}} }}\quad {\rm where}\quad {d_{ij}}^2 = {\left\| {{x_j} - {v_i}} \right\|^2}$$

The process starts by randomly choosing *c* objects that represent the centroids (means) of the *c* clusters. Membership values ${u_{ij}}$ are calculated based on the relative distance (i.e., Euclidean distance) of the object ${x_j}$ to the centroids. The centroids ${v_i}$ of the clusters are calculated after the memberships of all objects have been found. If the centroids at the previous iteration are identical to the centroids generated at the current iteration the process terminates (Maji & Pal, 2008).

As opposed to type-I fuzzy sets (i.e., fuzzy c-means), *type-II fuzzy sets* can model uncertainty since their membership functions are considered fuzzy (Mendel & John, 2002). They are created by firstly defining a type-I fuzzy set and then assigning *lower* and *upper* membership degrees to each element in order to construct the FOU (Footprint of Uncertainty) which encapsulates the uncertainty associated with the membership functions. A type-II fuzzy set can be defined as Tizhoosh (2005):

(6) $$\tilde A = \left\{ {\left( {x,{\mu _U}\left( x \right),x,{\mu _L}\left( x \right)} \right)|\forall x \in X,{\mu _L}\left( x \right) \le \mu \left( x \right) \le {\mu _U}\left( x \right),\mu  \in \left[ {0,1} \right]} \right\}$$

where ${{\rm{\mu}}_L}$ and ${{\rm{\mu}}_U}$ represent the *lower* and *upper* membership degrees of the initial membership function μ, respectively, and are defined as follows (Tizhoosh, 2005):

(7) $${{\rm{\mu}}_L}\left( x \right) = {\left[ {{\rm{\mu}} \left( x \right)} \right]^{\rm{\alpha}} }$$

(8) $${{\rm{\mu}} _U}\left( x \right) = {\left[ {{\rm{\mu}} \left( x \right)} \right]^{\textstyle{1 \over {\rm{\alpha}} }}}$$

where ${\rm{\alpha}} \in \left( {1,\infty } \right)$. The range of values ${\rm{\alpha}} \in (1,2]$ are recommended to use for image data since ${\rm{\alpha}}\ {\rm \gg }\ 2$ is usually not meaningful for such data (Tizhoosh, 2005). In this work α is set to $2$: ${{\rm{\mu}}_L}\left( x \right) = {\left[ {{\rm{\mu}} \left( x \right)} \right]^2}$ and ${{\rm{\mu}}_U}\left( x \right) = {\left[ {{\rm{\mu}} \left( x \right)} \right]^{0.5}}$.

The measure of *ultrafuzziness* (linear index of fuzziness) ${{\rm{\gamma}} ^\~}$ for an $M \times N$ image subset ${A^~} \subseteq X$ with $L$ gray levels $g \in [0,L - 1]$, histogram $h(g)$, and the membership function ${{\rm{\mu}}_{{A^~}}}\ (g)$, can be defined as Tizhoosh (2005):

(9) $${{\rm{\gamma}} ^~} = \displaystyle{1 \over {\rm MN}}\sum\limits_{g = 0}^{L - 1} h(g) \times [{{\rm{\mu}}_U}(g) - {{\rm{\mu}}_L}(g)]$$

where ${{\rm{\mu}}_U}(g)$ and ${{\rm{\mu}}_L}(g)$ are defined in Eqs. (7) and (8), respectively.

The edge ambiguity global estimation is provided by the *ambiguity threshold* (τ). Algorithm 1 depicts the algorithm used for calculating the ambiguity threshold based on type-II fuzzy sets and the measure of ultrafuzziness (Tizhoosh, 2005).

The membership function we use in this work is the *S-function* (Eq. (10)) since it enhances the contrast of the fuzzy image (represented in terms of its membership values) and reduces the amount of ultrafuzziness (Sladoje, Lindblad & Nystrom, 2004).

(10) $$S({\rm{\mu}} ;a,b,c) = \left\{ {\matrix{ {0,} &  {{\rm{\mu}} \le a} & \cr {\displaystyle{1 \over 2}{{\left( {\displaystyle{{{\rm{\mu}} - a} \over {b - a}}} \right)}^2},} &  {a < {\rm{\mu}} \le b} & \cr {1 - \displaystyle{1 \over 2}{{\left( {\displaystyle{{{\rm{\mu}} - c} \over {c - b}}} \right)}^2},} &  {b < {\rm{\mu}} \le c} & \cr {1,} &  {{\rm{\mu}} > c} & \cr } } \right.$$

where $a = 0$, $c = 1$, $b = \displaystyle{{a + c} \over 2} = 0.5$ (crossover point).

Different attempts have been made to measure the ambiguity threshold. However, such attempts suffer from limitations that we try to overcome in our approach. For instance, in Sladoje, Lindbald & Nyström (2011) in order to find the threshold a model of the membership function is found and the threshold is calculated within an α-cut, such that the α-cut value is manually and heuristically chosen rather than in a systematic way as proposed in our approach. The choice of the most appropriate threshold using this method is thus very difficult. In Otsu’s method (Otsu, 1979) on the other hand, when calculating the threshold the histogram must be unimodal and does not take into account the level of fuzziness. By following the image thresholding algorithm proposed in Algorithm 1, we overcome those aforementioned limitations in calculating the ambiguity threshold.

**Algorithm 1:** Measuring the ambiguity threshold.

1 Initialize the value α and determine the shape of the membership function
2 Find the image histogram
3 Initialize the position of the membership function, and shift it along the range of the gray-level values
4 At each position (gray-level value *g*), find the upper and lower membership values μ*_U_*(*g*) and μ*_L_*(*g*) respectively
5 Using Eq. (9), find the amount of ultrafuzziness at each position
6 Determine the position *g*_pos_ that has the maximum ultrafuzziness, and use this value to threshold the image (*T* = *g*_pos_)

In fuzzy clustering, the minimization of the function *J* (given in Eq. 2) leads to partitions characterized by the membership degree matrix. A *defuzzification* step is thus required to obtain the final segmentation. While usually the data (pixels) gets affected to the class with the highest membership degree, in skin lesion images such approach might not give appropriate results as lesion borders are sometimes not clearly defined.

**Figure 5:**
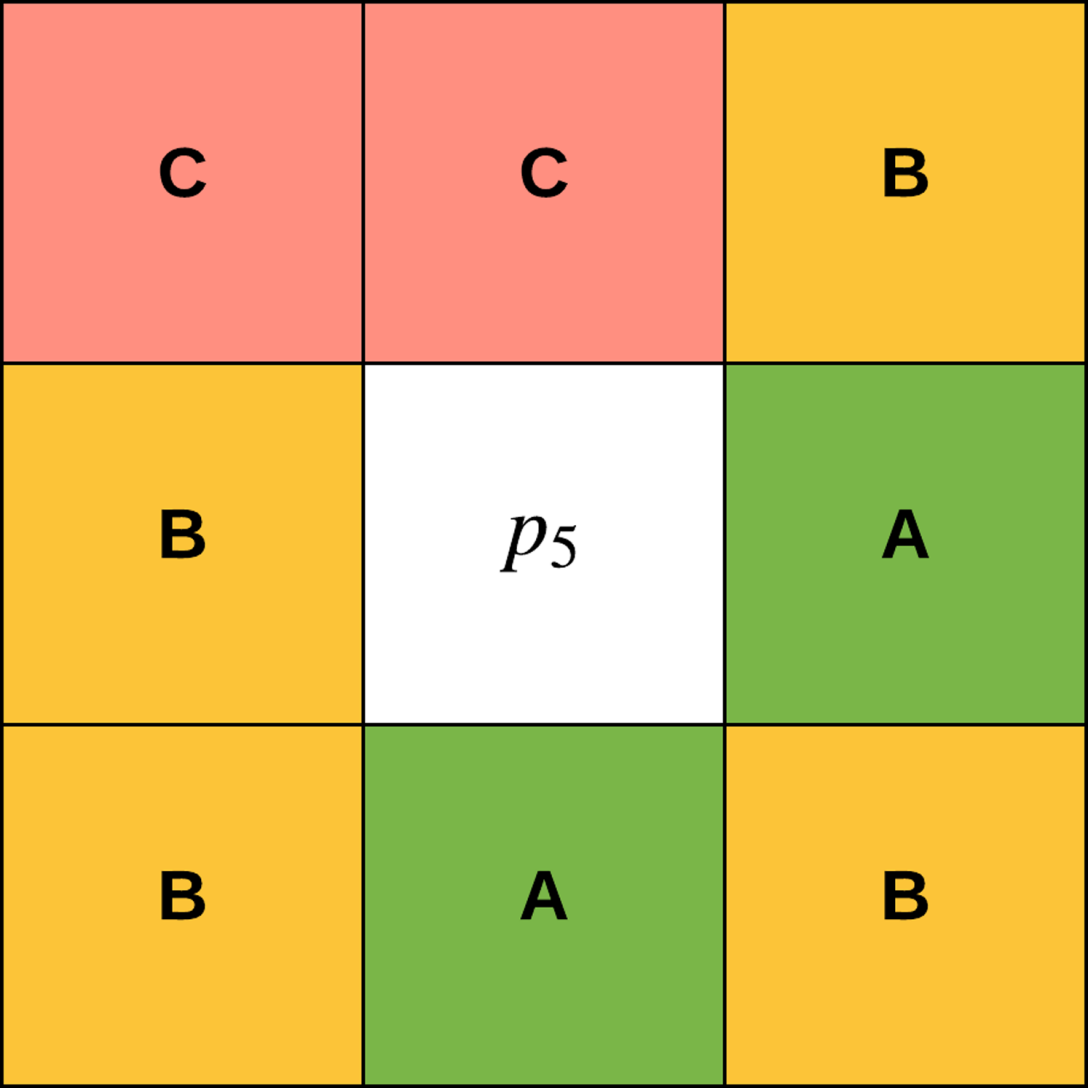
Weak pixel *p*5 will be assigned to cluster B.

The concept of *gradual focusing* (Fig. 4), inspired by the human visual perception and introduced by Boujemaa et al. (1992), proceeds in two steps: (i) membership values are compared with the ambiguity threshold τ to reveal the most ambiguous pixels which are considered to have a weak membership degree, from those that possess a high membership degree in order to represent the coarse image information and locate the inner parts of the regions. Ambiguous pixels are those that have a membership value smaller than τ (ii) Weak pixels are affected to the appropriate cluster with regards to their spatial context. The notion of local ambiguity for a given pixel is thus introduced by considering a spatial criterion describing the neighborhood. The whole image has to be explored to deal with all the ambiguous pixels; *Linear sweeping* is a method that can be used in such situation where the image pixel is affected to the major cluster of its neighbors. For instance, the weak pixel ${p_5}$ in Fig. 5 evaluated against its neighbors in a $3 \times 3$ window will be affected to cluster $B$. If the cluster frequency is equal for each cluster (class) around the weak pixel, the pixel will be assigned to the original cluster it was classified to belong to. In other words, if we have more than one major cluster around the weak pixel, the assigned cluster to the pixel will be the one to which the pixel has the highest membership degree (i.e., the defuzzification step carried out by FCM). This process continues until all the weak pixels are treated.

To make the edges of the segmentation result sharper for better edge detection, we use an edge-preserving image smoothing approach proposed in Cai, Xing & Xu (2017) which basically removes texture at any level without distorting edges through the use of a *local regularization* named Relativity-of-Gaussian (RoG) on which a *global optimization* is applied to identify potential edges at different scales. In other words, different scale edges are defined using different Gaussian kernels to preserve important structures with high resolution; edges that possess similar patterns in their neighbors would show more similar direction gradients. A global optimization function is subsequently defined to smooth the edges at different scales.

### Skin lesion border detection

After extracting the skin lesion using the process described in the previous section, we need to detect the lesion’s border as a prerequisite for measuring the border irregularity. For this task, we utilize the Canny edge detector (Canny, 1986) to detect the edges from the segmented image due to its robust ability in detecting edges especially in high-noise conditions (Vadivambal & Jayas, 2015), its ability to come up with the best trade-off between edge detection and localization, and provides information about edge strength (the magnitude of the gradient of the Gaussian smoothed image) (Bigler, 1996). Canny edge detection is carried out in four steps: (i) smooth the image using Gaussian filtering (ii) calculate the magnitude and direction of the image gradient (iii) non-maximum suppression (iv) set image threshold and connect the edge (Feng, Zhang & Wang, 2017).

In *Gaussian smoothing* the image is smoothed by a 1-D Gaussian function in order to remove noise before the edge detection process. Assuming that $f(x,y)$ is a grayscale image, the filtered image $I(x,y)$ can be expressed as:

(11) $$G\left( x \right) = \displaystyle{{exp\left( {\displaystyle{{ - {x^2}} \over {2{{\rm{\sigma}} ^2}}}} \right)} \over {2{\rm{\pi}} {{\rm{\sigma}} ^2}}}$$

(12) $$I\left( {x,y} \right) = \left[ {G\left( x \right)G\left( y \right)} \right] \times f\left( {x,y} \right)$$

where σ refers to the standard deviation (size) of the Gaussian filter $G(x)$, and controls the smoothing degree of the filter (Feng, Zhang & Wang, 2017).

The next step in Canny edge detection is the selection of a $2 \times 2$ neighboring area where extreme changes in grayscale occur (edge) to obtain the magnitude and direction of the *gradient*. The first order derivative on $X$ and $Y$ directions can be found using the following equations:

(13) $$M\left( {x,y} \right) = \sqrt {R_x^2\left( {x,y} \right) + R_y^2\left( {x,y} \right)}$$

(14) $$D\left( {x,y} \right) = {\rm arctan}\left[ {\displaystyle{{{R_y}\left( {x,y} \right)} \over {{R_x}\left( {x,y} \right)}}} \right]$$

(15) $${R_x} = \displaystyle{{\left( { - {S_1} + {S_2} - {S_3} + {S_4}} \right)} \over 2}$$

(16) $${R_y} = \displaystyle{{\left( {{S_1} + {S_2} - {S_3} - {S_4}} \right)} \over 2}$$

where $M(x,y)$ is the image gradient *magnitude* and $D(x,y)$ is the image gradient *direction*. ${R_x}$ and ${R_y}$ are the *X* and *Y* derivatives at a specific point, respectively. ${S_1}$, ${S_2}$, ${S_3}$, and ${S_4}$ are the pixel values of the image at locations $(x,y)$, $(x,y + 1)$, $(x + 1,y)$, and $(x + 1,y + 1)$, respectively (Feng, Zhang & Wang, 2017).

*Non-maxima suppression* then effectively locates the edge and suppresses the occurrence of false edges. A $3 \times 3$ neighboring area is used to compare a pixel with its two adjacent pixels along the gradient direction. If the magnitude of the pixel $M(i,j)$ is larger than the magnitude of the two adjacent pixels, the pixel will be marked as an edge point candidate, otherwise it will not be considered as an edge point (Feng, Zhang & Wang, 2017).

The final step is to *set the threshold and connect the edge* where the Canny edge detector uses both low and high thresholds to segment the image that resulted from the previous step (non-maxima suppression). If the gradient of the edge comes between the low and high thresholds, we analyze if any point around the pixel that is greater than the high threshold exists: if a point exists it is considered an edge point, a non-edge point otherwise. If the gradient of the edge is greater than the high threshold on the other hand, the pixel will be marked as a candidate edge point. The edge points of the candidate edge that are attached to the edge will be marked as edge points, obtaining the edge image and reducing the noise effects on the edge in the edge image (Feng, Zhang & Wang, 2017).

### Skin lesion border irregularity

After detecting the skin lesion border we need to measure the border’s irregularity which represents the B feature of the ABCD rule. For this task, we combine fractal dimension with both Zernike moments and convexity that would together serve as an *objective* quantitative measure of border irregularity, especially when many of the signs that the clinician relies on in diagnosis involve *subjective* judgment. This applies to visual signs such as border irregularity (Claridge et al., 1992). The inter-observer for skin lesion border did not also show sufficient reproducibility (*k*-value: 0.22) (Argenziano et al., 2003). In fact, it has been shown that both clinicians and patients find it hard in agreeing upon whether a naevus border is considered irregular or not (Claridge et al., 1992). Such measure could thus aid in improving the diagnostic accuracy.

**Figure 6:**
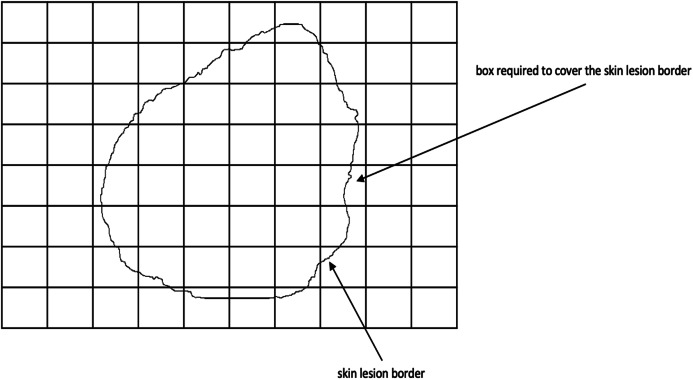
The box-counting method where 22 boxes are required to cover the skin lesion border.

Fractal dimension has been used in characterizing skin lesion border irregularity as in Claridge et al. (1992), Ng & Lee (1996), and Piantanelli et al. (2005). Fractal geometry (Mandelbrot, 1982) describes the space-filling capacity of irregular borders which is considered size independent and does not require any smoothing operations of irregular borders for measurement to be possible (Cross et al., 1995), meaning that structures don’t need to possess a perfect geometric shape. The *fractal dimension* is a mathematical parameter that can quantify the irregularity (roughness or smoothness) of a skin lesion border via an objective observer-independent value. It is related to the complexity of the shape associated with the border such that a higher fractal dimension would stand for a higher degree of complexity of the analyzed pattern. In a 2-dimensional system a straight line will have a fractal dimension of *one*, and more complicated lines (having fractal properties) will have larger dimensions (Falconer, 1990). In general, fractal objects are those whose ratios are not whole numbers but fractions. This leads us to conclude that if the irregular borders of melanoma have fractal properties then they would be described more accurately by fractal dimension than Euclidean measures (i.e., perimeter) (Cross & Cotton, 1992). In this article, we are going to use the *box-counting* method (Feder, 1992) for estimating the fractal dimension of the skin lesion border, defined as:

(17) $$D = \mathop {\lim }\limits_{{\rm{\varepsilon}} \to 0} \displaystyle{{\log N\left( {\rm{\varepsilon}} \right)} \over {\log \left( {\displaystyle{1 \over {\rm{\varepsilon}} }} \right)}}$$

where $D = [1,2]$ is the box-counting fractal dimension of the skin lesion border, ${\rm{\varepsilon}} > 0$ is the side (edge) length of the box, and *N* is the smallest number of boxes of side length ${\rm{\varepsilon}} needed to completely cover the skin lesion border (Fig. 6). The fractal dimension is the *slope* in the $\log N\left( {\rm{\varepsilon}} \right)/\log \left( {\textstyle{1 \over {\rm{\varepsilon}} }} \right)$ graph.

To demonstrate a fractal with a fractal dimension in the range $[1,2]$, the *Koch curve* can be used which is formed in multiple steps, such that in the first step a straight line is divided into three segments and the middle part is replaced by two segments with an equal length. Each straight segment in the subsequent steps is divided into three parts with the middle part of each step replaced by two parts. A Koch curve is formed when the process is carried out infinitely (Li et al., 2003).

The lower the value $D$ the straighter and smoother the skin lesion border, and vice versa. Melanoma borders, due to their irregularity, are more similar to fractals (i.e., Koch snowflake which is generated based on the Koch curve, such that the first step starts with an equilateral triangle (Li et al., 2003)) and are expected to have a higher fractal dimension than regular-boundary naevi. For instance, in Cross et al. (1995) it was found that the fractal dimension of all lesions are greater than the topological dimension (i.e., one), which indicates that there exists a fractal element in their structure.

Although the fractal dimension *D* provides values consistent with the rules normally used in clinical practice, in the aspect that *D* values significantly increase in melanoma lesions as compared to benign lesions, using *D* as a single parameter in distinguishing skin lesion border irregularity could be limited. Thus, combining it with other parameters should be considered (Piantanelli et al., 2005). The parameter we will combine with fractal dimension is a shape descriptor called *Zernike moments*.

Zernike moments are orthogonal moments, which means that no redundant or overlapping information exist between the moments, and are based on Zernike polynomials. They are invariant to rotation and are thus ideal for describing the shape characteristics of objects (i.e., skin lesions) (Tahmasbi, Saki & Shokouhi, 2011; Oluleye et al., 2014). Let $(m,n)$ be a pair representing the Zernike polynomial order and the multiplicity (repetition) of its phase angle, respectively. The Zernike moment can then be defined as Tahmasbi, Saki & Shokouhi (2011):

(18) $${V_{nm}}\left( {{\rm{\rho}} ,{\rm{\theta}} } \right) = {R_{nm}}\left( {\rm{\rho}} \right){e^{im {\rm{\theta}} }}, {\rm{\theta}} \le 1$$

where

(19) $${\rm{\rho}} = \sqrt {{x^2} + {y^2}} ,$$

(20) $${\rm{\theta}} = {\rm arctan}\left( {\displaystyle{y \over x}} \right)$$

(21) $${R_{nm}}\left( {\rm{\rho}} \right) = \sum\limits_{a = 0}^{\textstyle{{\left( {n - \left| m \right|} \right)} \over 2}} {\left( { - 1} \right)^a}\displaystyle{{\left( {n - a} \right)!} \over {a!\left( {\displaystyle{{n + \left\lceil m \right\rceil } \over 2} - a} \right)!\left( {\displaystyle{{n - \left\lceil m \right\rceil } \over 2} - a} \right)!}}{{\rm{\rho}} ^{n - 2a}}$$

ρ is the image pixel radial vector, θ is the angle between that vector and *x*-axis, and ${R_{nm}}$ is the Zernike polynomial, which is an orthogonal polynomial equation over a circular space; polynomials are a function of the Cartesian coordinates $(x,y)$ on the unit disc that are commonly expressed in terms of polar coordinates. In other words, the Zernike polynomial is defined in polar coordinates $({\rm{\rho}} ,{\rm{\theta}} )$ on a circle of unit radius $r < 1$. Zernike moment features describe the similarity of an input image to a set of Zernike polynomials. For an image $f({x_i},{y_i}):1 \le i \le M,1 \le j \le N$, the Zernike moment ${Z_{nm}}$ can be calculated as Oluleye et al. (2014):

(22) $${Z_{nm}} = \displaystyle{{n + 1} \over \rm{\pi} }\sum\limits_{i = 1}^M \sum\limits_{j = 1}^N {V_{nm}}\left( {{x_i},{y_j}} \right)f\left( {{x_i},{y_j}} \right)$$

where $m = 0,1,2,3,\ldots,\infty$ is the order of the Zernike polynomial, and $n$ is the multiplicity of the phase angles in the Zernike moment, ${x^2} + {y^2} \le 1$. Zernike moments produce a 25-value vector (order *n* = 8) as a description of the skin lesion contour.

Convexity can be used to characterize the skin lesion border shape and irregularity (Rosin, 2009; Lee, McLean & Stella, 2003; Do et al., 2018). It is the ratio between the perimeter (the number of points/length of the boundary) of the convex hull of the skin lesion (the smallest convex polygon that surrounds all of the skin lesion pixels) and the skin lesion perimeter. It shows the amount by which the object differs from the convex object. The convexity for the convex object evaluates to 1, and is less than 1 for non-convex objects (i.e., irregular skin lesion borders).

**Figure 7:**
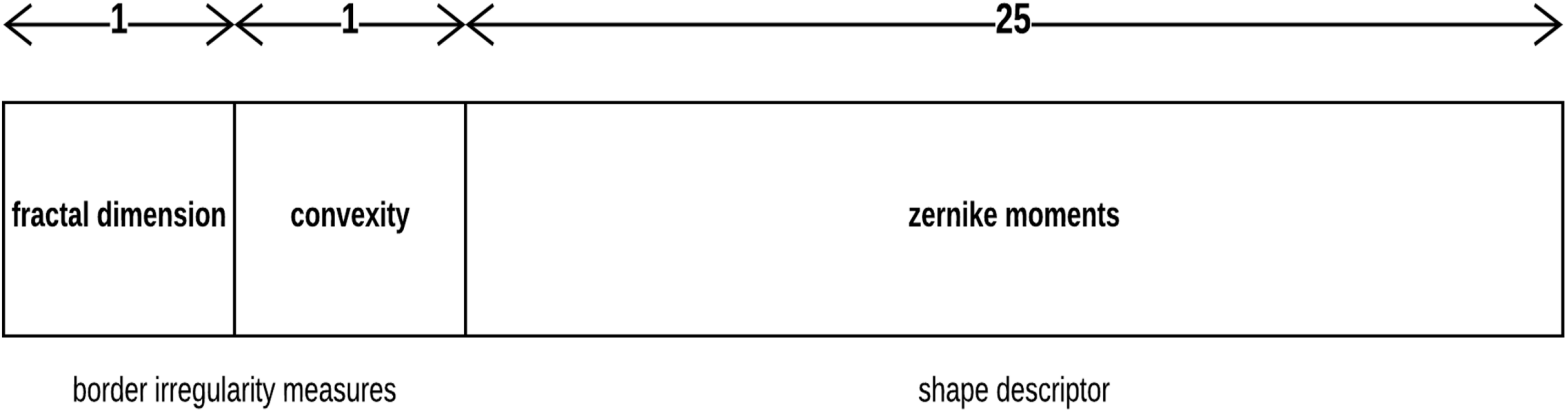
Skin lesion feature vector.

Each skin lesion is now represented by a 27-value vector as depicted in Fig. 7 below.

### Convolututional neural networks

**Figure 8:**
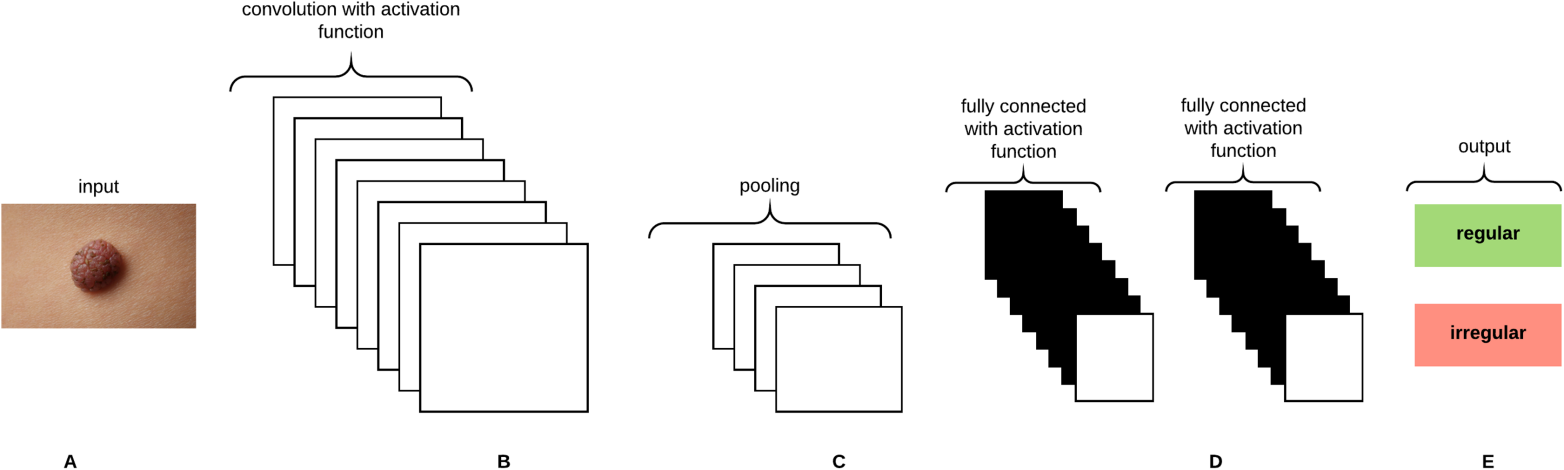
A CNN architecture composed of five layers. (A) Input image. (B) Convolution layer. (C) Max-pooling layer. (D) Fully-connected layers. (E) Classification result.

Convolutional Neural Networks (CNNs) are analogous to Artificial Neural Networks (ANNs) in that they consist of neurons that self optimize through learning, such that each neuron would receive an input and perform an operation (i.e., scalar product) followed by a non-linear function. The neurons in the CNN are organized into height, width, and depth. Unlike ANNs, neurons within any given layer will connect to a small region of the preceding layer (O’Shea & Nash, 2015). CNNs are thus considered a specialized type of neural networks that process data having a grid-like topology (i.e., images can be thought of as a 2D grid of pixels) (Goodfellow, Bengio & Courville, 2016). CNNs are emerged from the study of the brain’s visual cortex and have been used in image recognition since the 1980s. With the increase in computational power and amount of training data, CNNs are able to achieve superhuman performance on some complex visual tasks. The *convolutional layer* is considered the most important building block of the CNN. Neurons in the first convolutional layer are connected to pixels in their receptive fields as opposed to each pixel in the input image. Neurons in the second layer are thus connected to neurons that are located within a small rectangle in the first layer. Such architecture allows the network to focus on low-level features in the first hidden layer and then assemble them into higher level features in the next hidden layer, and so on, rendering CNNs to work well in image recognition tasks (Géron, 2017). A neuron located in row *i* and column *j* of the feature map *k* in a given convolutional layer *l* is connected to the outputs of the neurons in the previous layer *l-1* located in rows *i* to $i + {f_h} - 1$ and columns *j* to $j + {f_w} - 1$, where ${f_h}$ and ${f_w}$ are the height and width of the receptive field, respectively. The neuron’s weights (*filters* or *convolutional kernels*) can be represented as a small image of the size of a receptive field. A layer full of neurons using the same filter will give a *feature map* that highlights the areas in an image that are most similar to the filter. During the training process, a CNN attempts to find the most useful filters for the task and learns to combine them into more complex patterns. All the neurons share the same parameters (weights and bias) within one feature map. CNNs are composed of three types of layers: convolutional layers, pooling layers, and fully-connected layers. Stacking these layers together forms the CNN architecture as depicted in Fig. 8.

### Gaussian Naive Bayes

The Naive Bayes classifier is a probabilistic classifier that applies Bayes’ theory with strong (naive) independent assumption (i.e., independent feature model). The presence/absence of a particular feature of a class is not related to the presence/absence of any other feature. For instance, a skin lesion might be considered a melanoma if it has a larger fractal dimension and a smaller convexity. If those features depend on each other or upon the existence of other features (i.e., Zernike moments), the naive Bayes classifier considers all those features to *independently* contribute to the probability that a skin lesion is considered a melanoma. An advantage of the naive Bayes classifier is that it only requires a small amount of training data to estimate the *means* and *variances* required for classification. The probability model of the classifier can be represented (using Bayes’ theorem) as:

(23) $$p\left( {C|{F_1},\ldots ,{F_n}} \right) = \displaystyle{{p\left( C \right)p\left( {{F_1},\ldots,{F_n}|C} \right)} \over {p\left( {{F_1}, \ldots ,{F_n}} \right)}}$$

The probability model is a conditional model over a dependent class variable *C* with a small number of classes conditional on feature variables ${F_1}$ to ${F_n}$. The numerator is equivalent to the joint probability model: $p(C,{F_1}, \ldots ,{F_n})$. Since the denominator does not depend on *C* and the values of the features ${F_i}$ are known, the denominator is considered constant. As each feature ${F_i}$ is conditionally independent of every other feature ${F_j}$ where $i \ne j$, the joint probability model can be written as:

(24) $$p\left( {C,{F_1},\ldots ,{F_n}} \right) = p\left( C \right)p\left( {{F_1}|C} \right)p\left( {{F_2}|C} \right)p\left( {{F_3}|C} \right)\ldots = p\left( C \right)\prod\limits_{i = 1}^n p\left( {{F_i}|C} \right)$$

In *Gaussian naive Bayes*, Gaussian distributions are used to represent the likelihoods of the features conditioned on the classes. Each feature can be defined by a Gaussian probability density function (having a Bell shape) defined as:

(25) $${F_i}{\rm \sim }N\left( {{\rm{\mu}} ,{{\rm{\sigma}} ^2}} \right)$$

where ${\rm{\mu}}$ is the mean, ${{\rm{\sigma}} ^2}$ is the variance, and:

(26) $$N\left( {{\rm{\mu}} ,{{\rm{\sigma}} ^2}} \right)\left( x \right) = \displaystyle{1 \over {\sqrt {2\rm\pi {{\rm{\sigma}} ^2}} }}{e^{ - \textstyle{{{{\left( {x - {\rm{\mu}} } \right)}^2}} \over {2{{\rm{\sigma}} ^2}}}}}$$

## Results and Discussion

**Figure 9:**
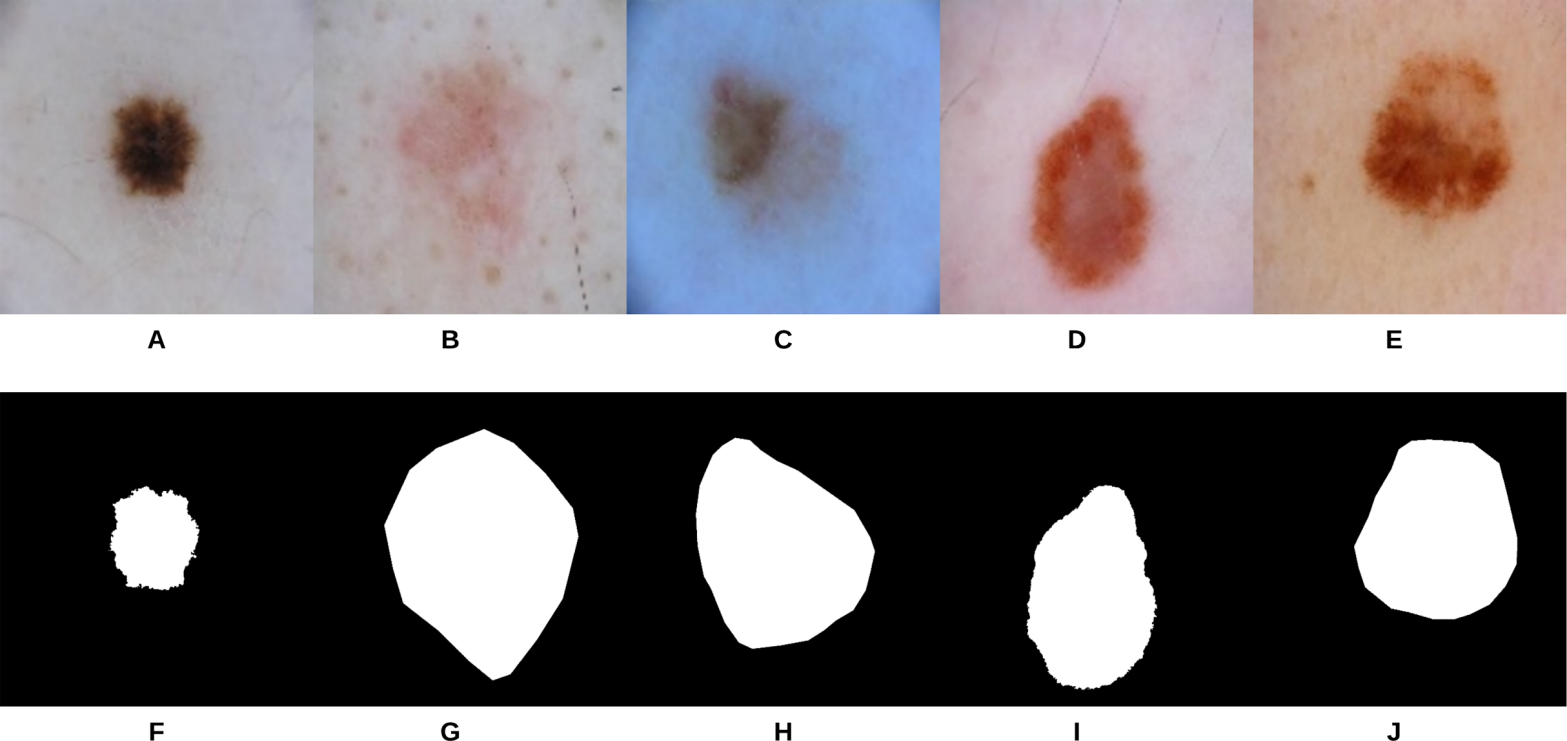
Samples of dermoscopy images (A–E) along with their corresponding groundtruth (F–J) used to train U-Net.

**Figure 10:**
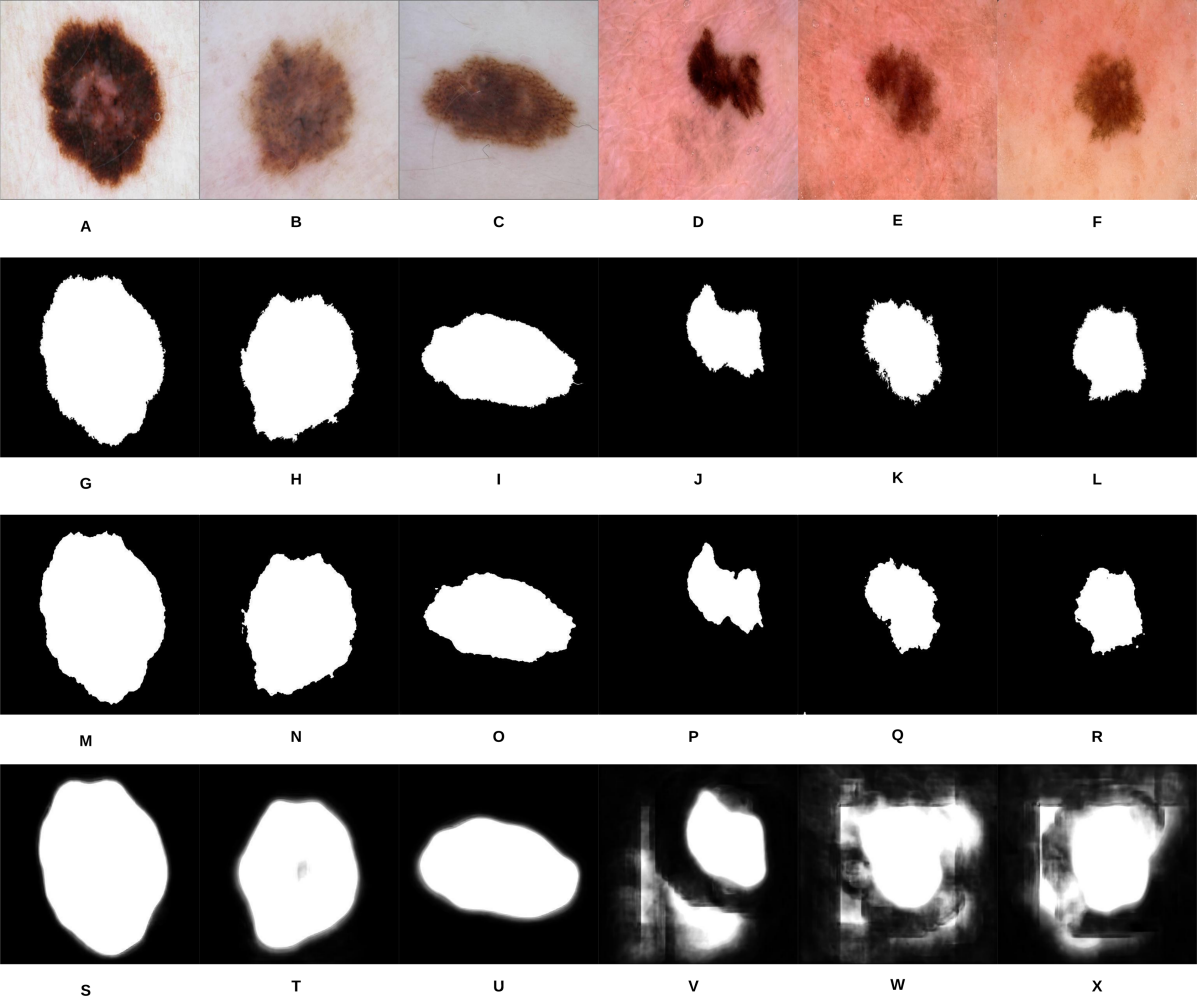
Samples of our proposed segmentation method and U-Net results, where (A–F) represent the original skin lesion images, (G–L) represent the groundtruth images, (M–R) are our method’s results, and (S–X) depict the U-Net results.

U-Net (Ronneberger, Fischer & Brox, 2015) is an end-to-end encoder-decoder network that has been firstly used in medical image segmentation. In our recent work, it has also been used in skin lesion segmentation in dermoscopic images (Ali, Li & Trappenberg, 2019; Ali et al., 2019). To compare our segmentation approach (i.e., gradual focusing) with U-Net, the two approaches were applied on 307 images from the “ISIC 2018: Skin Lesion Analysis Towards Melanoma Detection” grand challenge datasets (Codella et al., 2017a; Tschandl, Rosendahl & Kittler, 2018). The U-Net architecture was trained on 2,037 dermoscopy images along with their corresponding ground truth response masks (see Fig. 9 for some samples on the training images). Images used to train U-Net were resized to $512 \times 512$ pixels, and the model was trained for 20 epochs on a Tesla P100 GPU. Training the model took 115.3 min and testing it on the 307 images took 46 s. Examples of test images along with their corresponding groundtruth and segmentation results using the two approaches can be seen in Fig. 10.

As can be noticed from Fig. 10, our segmentation method is able to detect the fine structures of the skin lesion borders, as opposed to U-Net which lacks this ability. Detecting the fine structures is a crucial factor in determining the skin lesion border irregularity. Moreover, when the intensity between the background (skin) and skin lesion becomes closer (as in the last three images), U-Net produces noise in the segmentation results. The average Jaccard Index value of all the samples evaluates to 90.64% using our segmentation method, while using U-Net evaluates to 58.31%; in Jaccard Index the area of overlap *J* is calculated between the segmented binary image *S* and its ground truth *G* using the equation $J = \textstyle{{\left| {A \cap S} \right|} \over {\left| {A \cup S} \right|}} \times 100\%$, where the value $100\%$ means that the two values agree perfectly, while $0\%$ means that there is no overlap.

**Figure 11:**
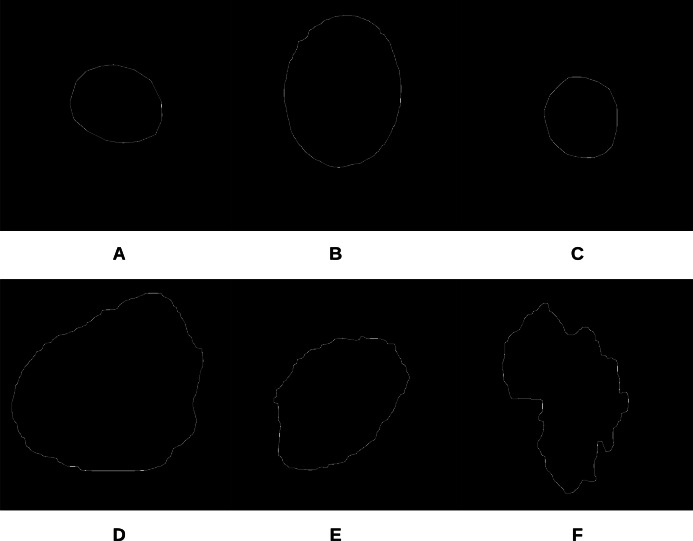
Samples of skin lesion border images used in the training phase and labeled by the dermatologist: (A–C) are regular edges and (D–F) are irregular edges.

To begin the process, 250 skin lesion borders extracted from skin lesion images from the “ISIC 2018: Skin Lesion Analysis Towards Melanoma Detection” grand challenge datasets were sent to a dermatologist (Dr. Sally O’Shea) to label as *regular* or *irregular*. Most of the images had an irregular border, summing up to 244 vs. 6 regular bordered images. Images are resized to $512 \times 512$ pixels. To make the most of the training data and to deal with the data imbalance, augmentation using some transformations has been applied (such as rotating, and flipping horizontally and vertically). 2,000 images were generated after the augmentation process, with each class (regular or irregular) having 1,000 images. This step was required for the training phase of our approach. Figure 11 shows some samples of the skin lesion border images used in the training phase.

**Figure 12:**
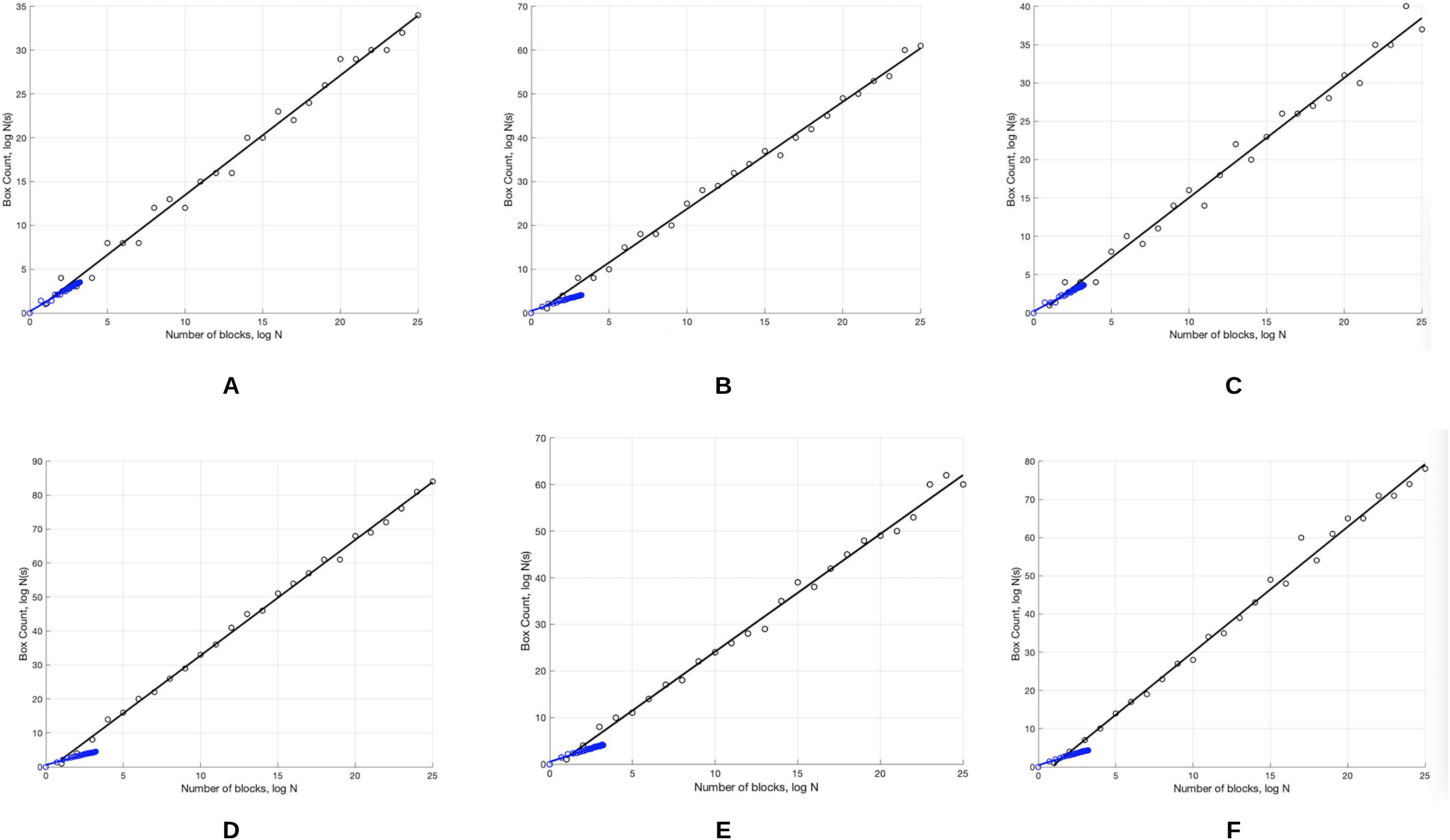
The log-log plots corresponding to the skin lesion borders shown in Fig. 11 where the fractal dimension values are determined from the slope of the plot. The fractal dimension values evaluate to: (A) 1.0394, (B) 1.1486, (C) 1.0679, (D) 1.2348, (E) 1.1481, (F) 1.2510.

**Figure 13:**
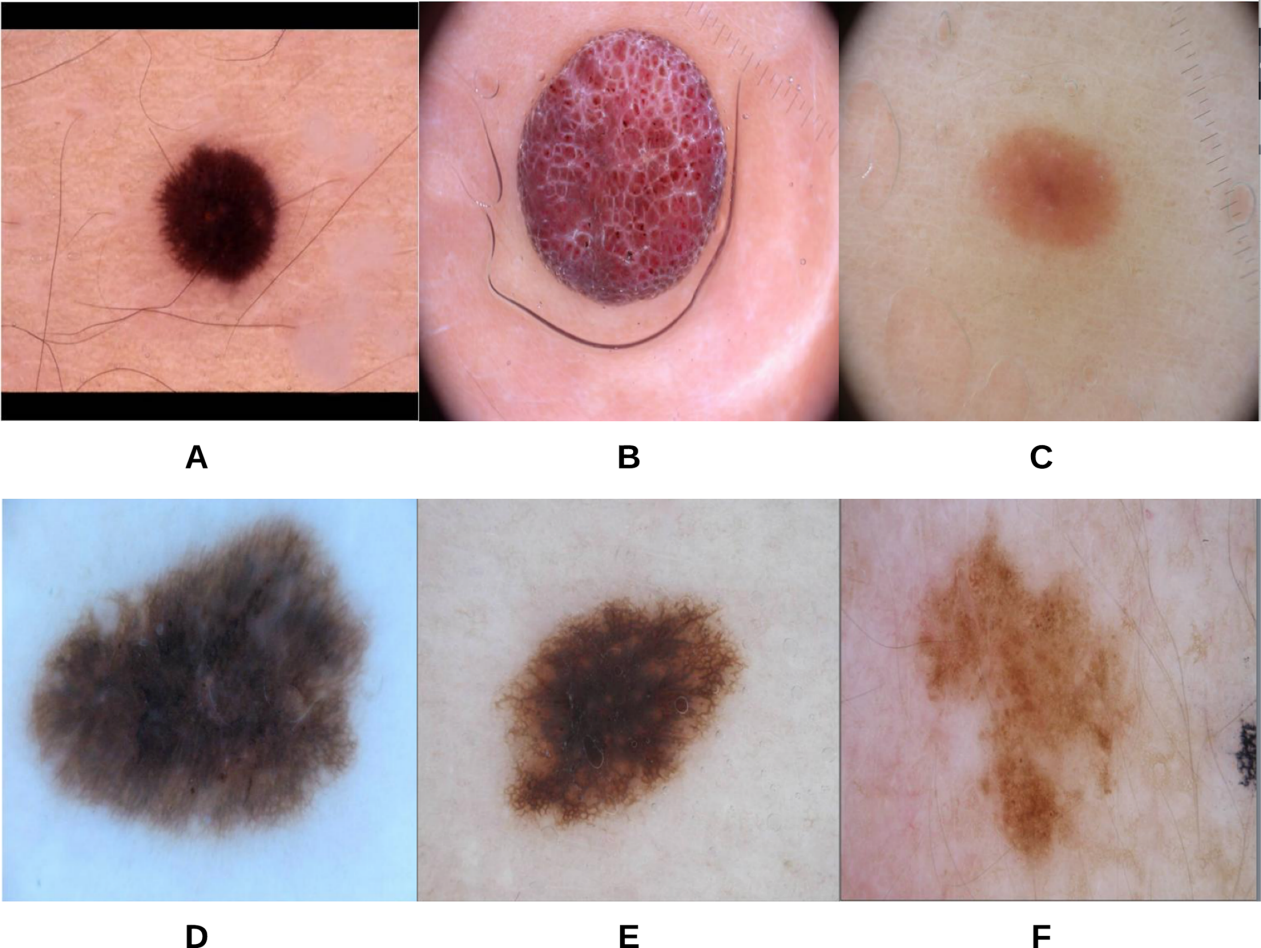
The original images (A–F) corresponding to the skin lesion borders shown in Fig. 11.

**Figure 14:**
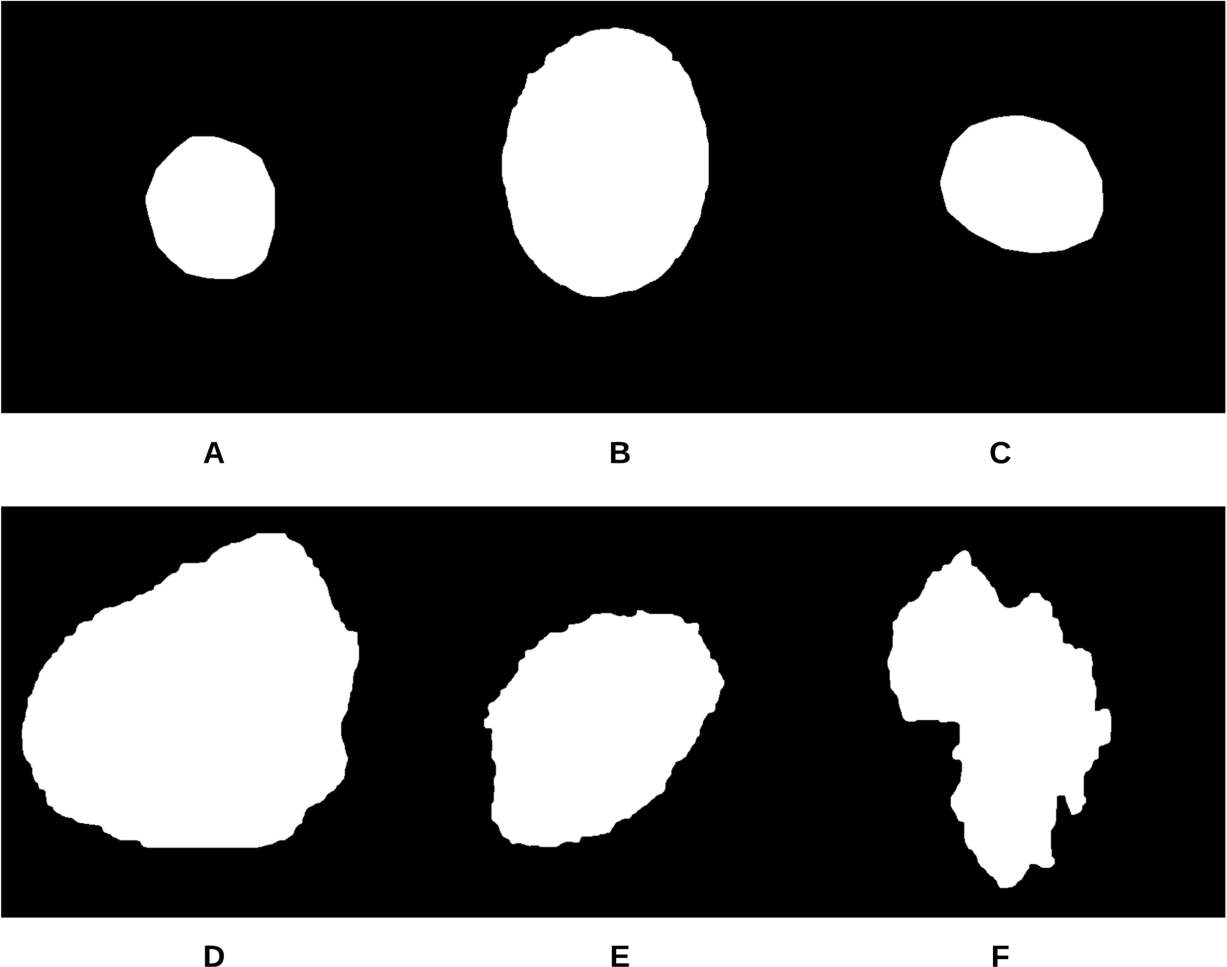
The smoothed segmented images (A–F) corresponding to the skin lesion borders shown in Fig. 11.

**Figure 15:**
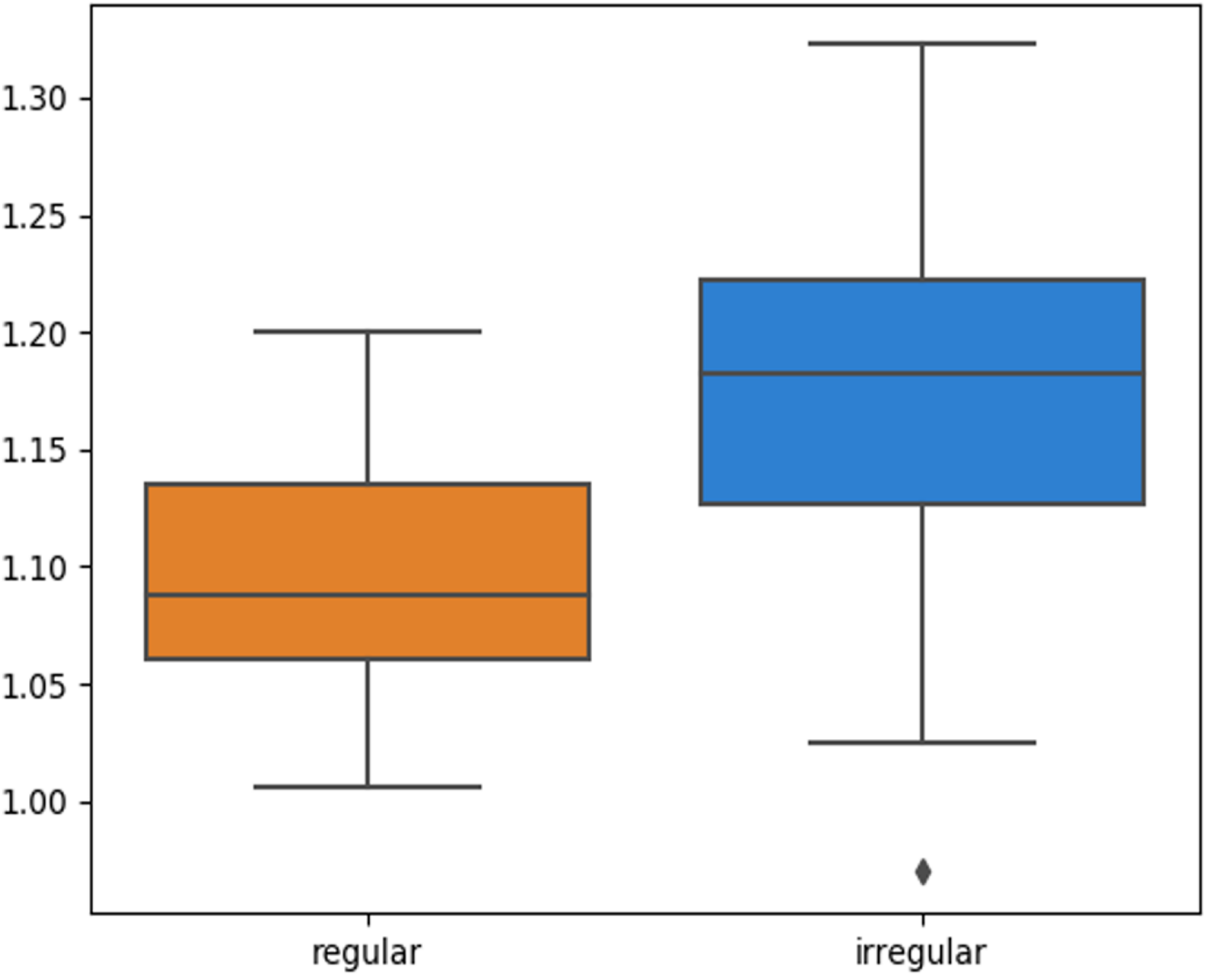
Box-and-whisker plot representing the fractal dimension distribution of the skin lesion borders (regular and irregular) in the training data.

**Figure 16:**
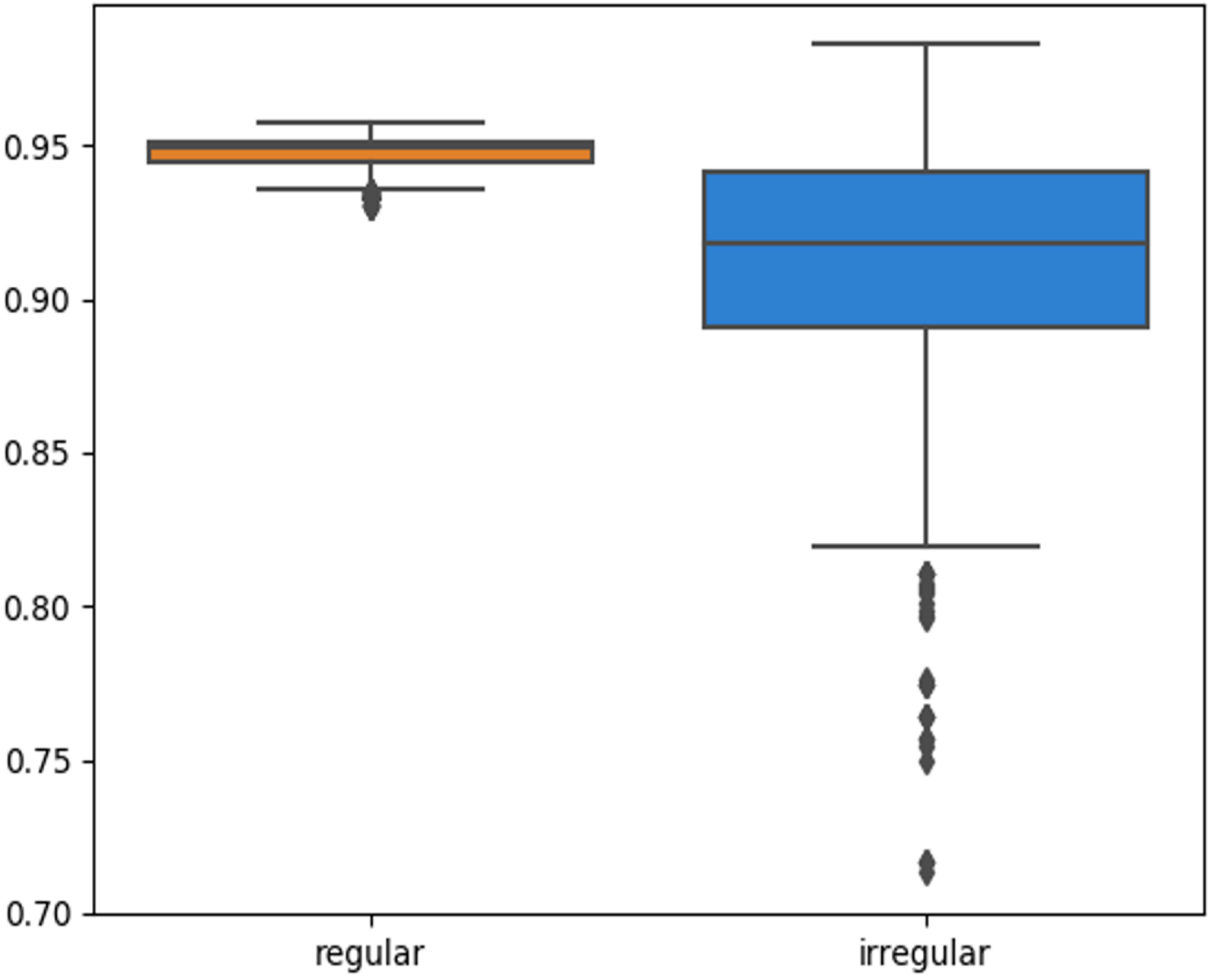
Box-and-whisker plot representing the convexity distribution of the skin lesion borders (regular and irregular) in the training data.

**Figure 17:**
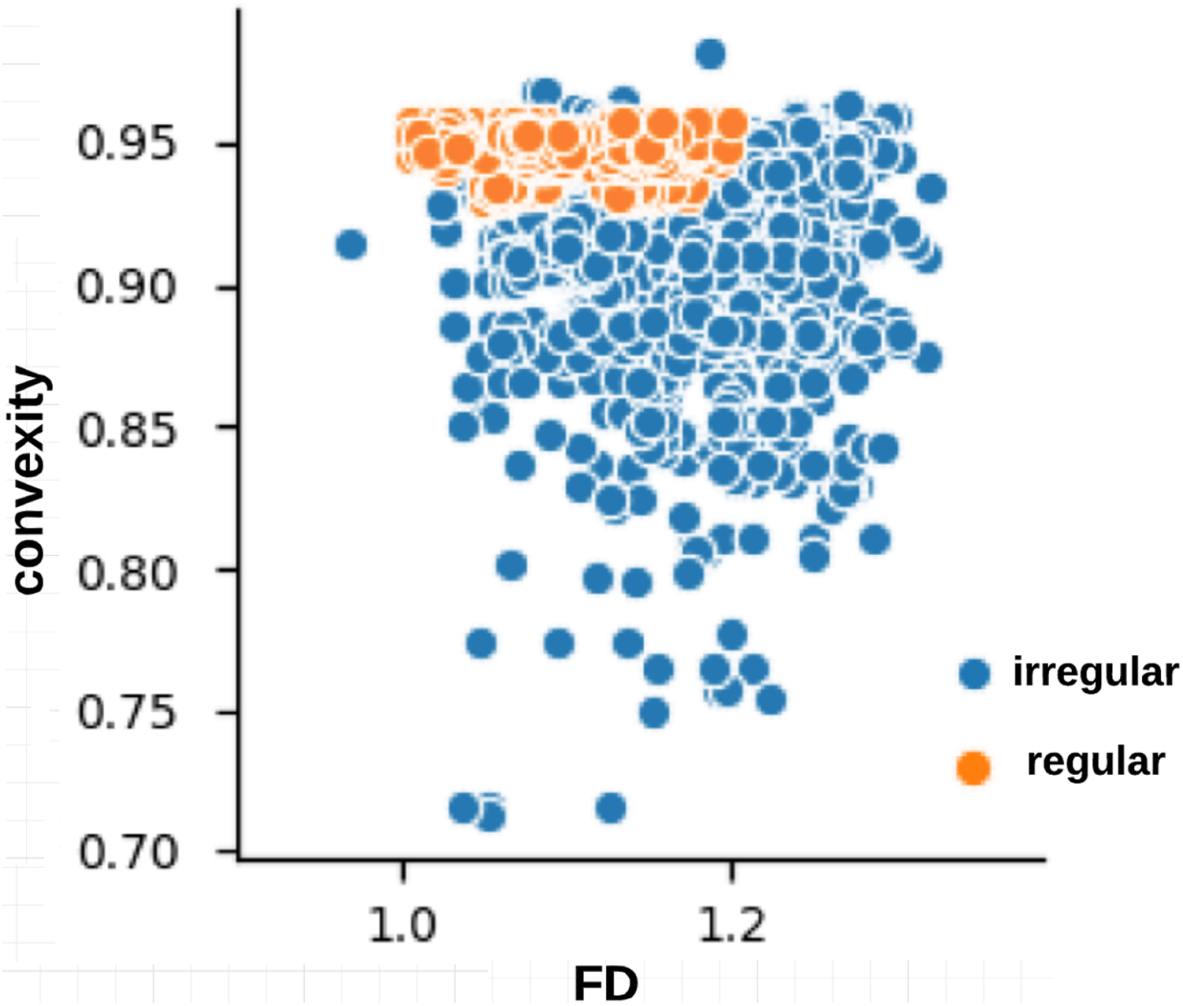
Relationship between fractal dimension and convexity values for regular (label:1) and irregular (label:0) skin lesion borders in the training dataset.

The skin lesion border irregularity step is then applied to the extracted borders, producing a 27-value vector of measures (Fig. 7) that together describe the irregularity inherent in each extracted border. Table 2 shows the extracted fractal dimension measures for the images shown in Fig. 11, and the log–log graphs of the corresponding images are depicted in Fig. 12 where the fractal dimension values are determined from the slope (the amount of change along the *y*-axis divided by the amount of change along the *x*-axis) of each plot. Convexity and Zernike moment values are extracted from the smoothed segmented images (the first 10 values of the 25-value Zernike moment vector are shown in the table). Figures 13 and 14 show the original and smoothed segmented results corresponding to the skin lesion borders shown in Fig. 11, respectively. The label column L in Table 2 is manually added and reflects the labeling made by the dermatologist. Figure 15 shows a box-and-whisker plot depicting the distribution of fractal dimension values for the regular and irregular skin lesion borders used in training the machine learning algorithms (classifiers). As can be noticed, the irregular skin lesion borders tend to move towards higher fractal dimension values (i.e., the more irregular the skin lesion border the higher the fractal dimension). Another plot is drawn in Fig. 16 to depict the distribution of convexity values, where irregular skin lesion borders tend to move away from the value 1 (less convex). Figure 17 depicts the relationship between the fractal dimension and convexity which shows that irregular borders (label:0) tend to have larger fractal dimension values and smaller convexity values, whilst regular borders (label:1) tend to have smaller fractal dimension values and larger convexity values.

**Table 2:** Border irregularity measures for the images presented in Fig. 11. FD, Fractal Dimension; C, Convexity; ZM, Zernike Moment; L, Label (regular: 1; irregular: 0).

Image	FD	C	ZM 1	ZM 2	ZM 3	ZM 4	ZM 5	ZM 6	ZM 7	ZM 8	ZM 9	ZM 10	L
A	1.0394	0.9573	0.3183	0.0004	0.0017	0.0027	0.0007	0.0051	0.0028	0.0046	0.0011	0.0009	1
B	1.1486	0.9354	0.3183	0.0001	0.0010	0.0028	0.0004	0.0047	0.0015	0.0043	0.0010	0.0008	1
C	1.0679	0.9482	0.3183	0.0003	0.0003	0.0028	0.0006	0.0039	0.0003	0.0045	0.0022	0.0012	1
D	1.2348	0.9208	0.3183	0.0006	0.0031	0.0017	0.0013	0.0048	0.0051	0.0029	0.0016	0.0022	0
E	1.1481	0.9006	0.3183	0.0010	0.0004	0.0014	0.0021	0.0013	0.0005	0.0025	19.0.0049	0.0032	0
F	1.2510	0.7747	0.3183	0.0001	0.0024	0.0023	0.0001	0.0057	0.0040	0.0027	0.0002	0.0089	0

The smoothed segmented images, skin lesion border images, and irregularity measures of the training data are used to train the CNN, which is composed of 5 convolutional layers, 5 max-pooling layers, and 2 dense layers. The convolutional layers use the ReLU activation function, the first dense layer uses the ReLU activation function, and the last dense layer uses the Sigmoid activation function. Adam is used as an optimization algorithm where the learning rate is set to 0.001. The CNN model is trained for 1 epoch on a Tesla P100 GPU; training for more epochs didn’t improve the training accuracy. Gaussian naive Bayes is trained on the irregularity measures on an Intel(R) Core(TM) i7-4770HQ CPU @ 2.20 GHz.

**Figure 18:**
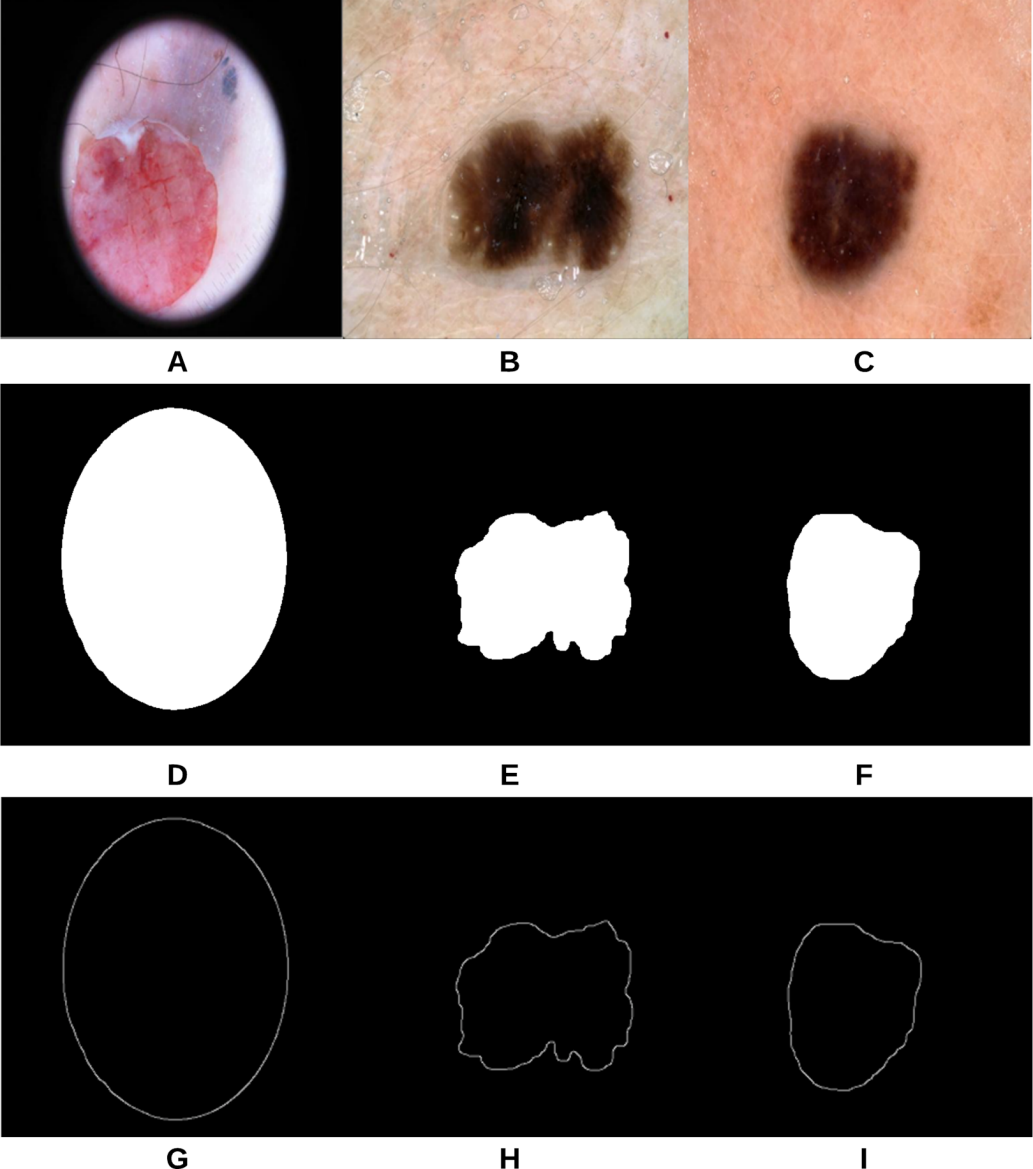
Samples of test images (A–C), smoothed segmented output (D–F) and their extracted skin lesion borders (G–I).

The proposed approach (Fig. 3) was applied on 47 randomly selected test images extracted from the “ISIC 2018: Skin Lesion Analysis Towards Melanoma Detection” datasets, provided that those images were not used in the training phase of the approach. For evaluation purposes to compare the results with a groundtruth, we asked the dermatologist to label the test images (the algorithms did not see before), resulting in 40 images being labeled as irregular and 7 images as regular. Figure 18 shows some samples of the test images, smoothed segmented results and their extracted skin lesion borders.

The test samples prediction probabilities of the two classes (irregular and regular) obtained using the training model of both the CNN and Gaussian naive Bayes are combined together (i.e., ensemble), resulting in a total prediction probability *P* calculated based on the following equation:

(27) $$P = \displaystyle{{{\rm CN}{N_{{p_1}}} \times Gn{B_{{p_1}}} + {\rm CN}{N_{{p_2}}} \times Gn{B_{{p_2}}}} \over 2}$$

where ${\rm CN}{N_{{p_1}}}$ and $Gn{B_{{p_1}}}$ are the prediction probabilities of the *first* class (i.e., irregular) resulting from the CNN and Gaussian naive Bayes, respectively. ${\rm CN}{N_{{p_2}}}$ and $Gn{B_{{p_2}}}$ are the prediction probabilities of the *second* class (i.e., regular) resulting from the CNN and Gaussian naive Bayes, respectively. After obtaining the *prediction probability* of each test sample using Eq. (27), a *threshold* is generated on those prediction probabilities to decide the final prediction (irregular or regular) according to Eq. (28) that takes into account all the prediction probabilities including the peak (maximum) probability.

(28) $$\rm threshold\quad = \quad \displaystyle{{{\rm max}\left( P \right) + {\rm mean}\left( P \right)} \over 2}$$

where *max*(*P*) is the maximum prediction probability value amongst all test prediction probabilities, and *mean* (*P*) is the mean (average) value of all test prediction probabilities. The final decision is eventually obtained using Eq. (29).

(29) $${\rm{Decision}} = \{ \matrix{{{\rm{regular}},} & {{P_i} < {\rm{threshold}})}  \cr{{\rm{irregular}},} & {{P_i} > {\rm{threshold}})}  \cr} $$

where ${P_i}$ is the prediction probability of test sample *i*.

**Figure 19:**
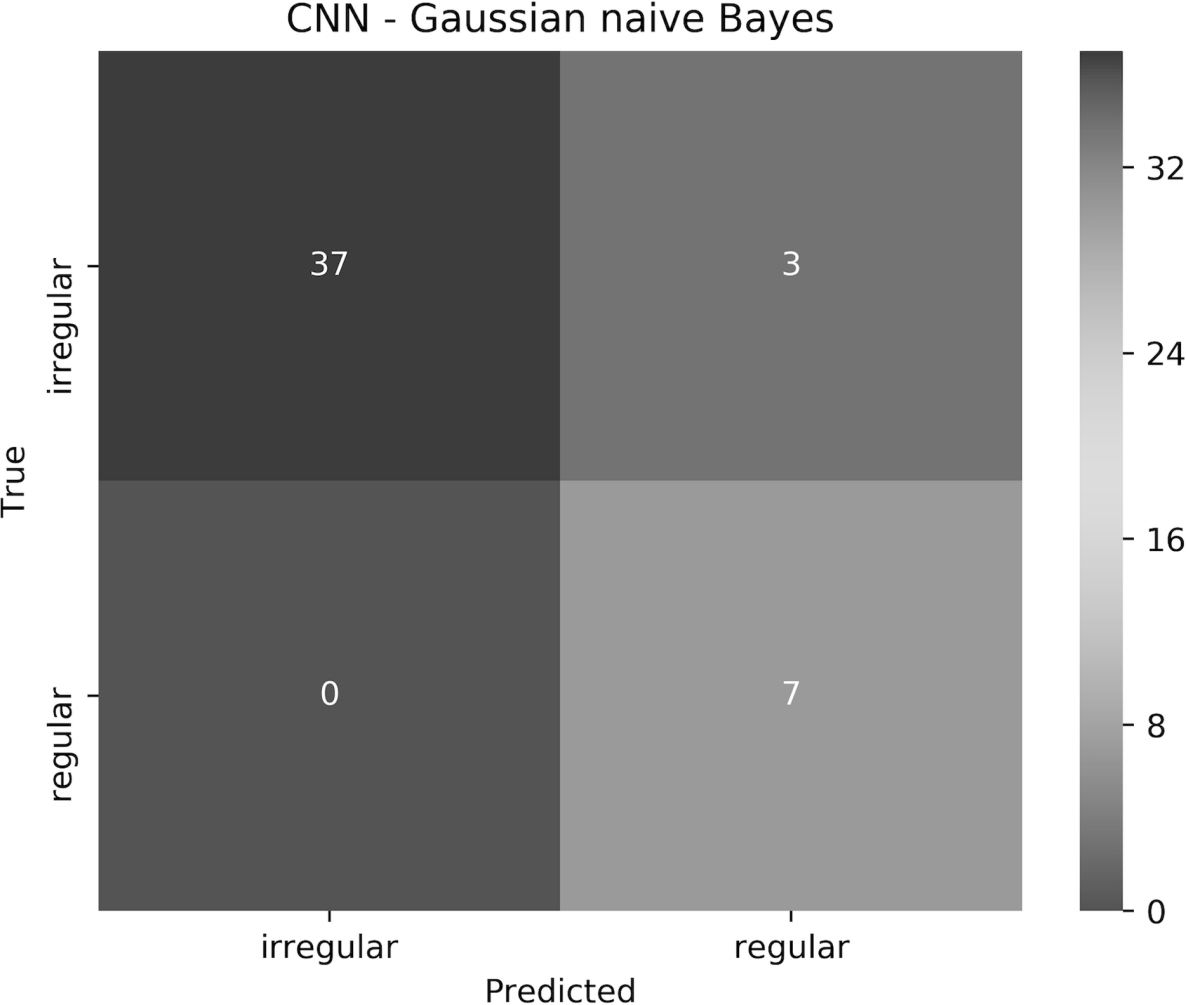
The confusion matrix of the test results obtained by the CNN—Gaussian naive Bayes ensemble.

The proposed approach resulted in 93.6% accuracy, where all the regular borders were predicted correctly, and 3 irregular borders were misclassified as regular. The elapsed time for training the CNN for 1 epoch and testing it on a Tesla P100 GPU evaluated to 7.1 min and 9.48 s, respectively. Training and testing using the Gaussian naive Bayes on an Intel(R) Core(TM) i7-4770HQ CPU @ 2.20 GHz together took 0.042 s. To understand the approach performance further (from different angles other than only accuracy), we have generated the confusion matrix shown in Fig. 19, in addition to finding the sensitivity, specificity, and F-score values, which resulted in 100%, 92.5% and 96.1%, respectively.

We believe that in our case False Positive (FP) is more important than False Negative (FN). In other words, if a patient had an irregular skin lesion border but was told (diagnosed) to have a regular border (FP), this might be life-threatening as opposed to classifying the patient to have an irregular skin lesion border while having a regular border (FN), which simply would send the patient for further investigation (i.e., biopsy). The approach works well in reducing FP and FN, with 3 misclassifications and 0 misclassifications, respectively. It should be emphasized that when evaluated separately, Gaussian naive Bayes and CNN result in 87.2% and 85.1% accuracy, respectively.

## Conclusion

An approach to automatically classify skin lesion border images into regular or irregular borders (i.e., the B feature of the ABCD rule) has been proposed.The developed segmentation method (the first stage of the approach) has been compared with U-Net and showed better accuracy in terms of Jaccard Index, in addition to its ability to detect fine structures better which are crucial to our irregularity detection task. An irregularity measure was also created which combines fractal dimension, convexity, and zernike moments. The irregularity measure along with the segmented image and border image were used to train the CNN, while the measure alone was used to train a Gaussian naive Bayes. The models generated from both networks were eventually combined together (ensemble) to test new images, and a threshold was created to determine the final classification decision from the test predictions. Results show that the approach achieves outstanding accuracy, sensitivity, specificity, and *F*-score results, reducing also False Positives (FPs) and False Negatives (FNs). The main research contributions in this work lie in the (i) proposition of an image segmentation approach that takes the ambiguous pixels into account by revealing and affecting them to the appropriate cluster in a fuzzy clustering setting (ii) proposing an objective quantitative measure for skin lesion border irregularity (iii) utilizing a CNN—Gaussian naive Bayes ensemble for predicting skin lesion border irregularity.

We understand that the process of labeling skin lesion border images into regular and irregular images is considered laborious and might involve a larger team for labeling out thousands of images, which we believe would improve the prediction results further. Adding more training samples with a more *balanced* dataset could also improve the results. This leads to our future endeavour of building a large dataset of regular and irregular border images which could facilitate the development of classification algorithms geared towards the B feature and provide an objective decision on the irregularity of the skin lesion border. Another motivation is to study different machine learning algorithms and networks that could reduce the training time as in the case with CNNs.

## Supplemental Information

10.7717/peerj-cs.268/supp-1 Supplemental Information 1 Sample of a training raw data.

10.7717/peerj-cs.268/supp-2 Supplemental Information 2 Sample of a testing raw data.

10.7717/peerj-cs.268/supp-3 Supplemental Information 3 Checks the neighbourhood of the ambiguous pixels in order to assign them to the appropriate cluster.

10.7717/peerj-cs.268/supp-4 Supplemental Information 4 Finds the fractal dimension of the image.

10.7717/peerj-cs.268/supp-5 Supplemental Information 5 Edge detection using canny edge detector.

10.7717/peerj-cs.268/supp-6 Supplemental Information 6 Returns the final classification based on the decision criteria.

10.7717/peerj-cs.268/supp-7 Supplemental Information 7 Finds the zernike moments and the convexity of an image.

10.7717/peerj-cs.268/supp-8 Supplemental Information 8 Image smoothing.

10.7717/peerj-cs.268/supp-9 Supplemental Information 9 The CNN architecture (training and testing).

10.7717/peerj-cs.268/supp-10 Supplemental Information 10 The gradual focusing approach proposed in the paper for image segmentation.

10.7717/peerj-cs.268/supp-11 Supplemental Information 11 Finds the threshold that distinguishes between the ambiguous and non-ambiguous pixels.

10.7717/peerj-cs.268/supp-12 Supplemental Information 12 The membership function (S-function) used in the ambiguity threshold calculation.

10.7717/peerj-cs.268/supp-13 Supplemental Information 13 The Gaussian naive Bayes classifier.

10.7717/peerj-cs.268/supp-14 Supplemental Information 14 Returns the ambiguous pixel.

## Additional Information and Declarations

### Data Availability

The following information was supplied regarding data availability: The code and raw data are available in the Supplemental Files.
